# Structural characterization of wax esters using ultraviolet photodissociation mass spectrometry

**DOI:** 10.1007/s00216-024-05434-2

**Published:** 2024-07-20

**Authors:** Barbora Kloudová, Vladimír Vrkoslav, Miroslav Polášek, Zuzana Bosáková, Josef Cvačka

**Affiliations:** 1 Institute of Organic Chemistry and Biochemistry of the Czech Academy of Sciences, Flemingovo náměstí 542/2, 160 00 Prague 6, Czech Republic; 2 Department of Analytical Chemistry, Faculty of Science, Charles University in Prague, Hlavova 2030/8, CZ-128 43, Prague 2, Czech Republic; 3 J. Heyrovský Institute of Physical Chemistry of the Czech Academy of Sciences, Dolejškova 2155/3, 182 23 Prague 8, Czech Republic

**Keywords:** Wax ester, UV photodissociation, Photochemistry, Double bond, Mass spectrometry

## Abstract

Wax esters play critical roles in biological systems, serving functions from energy storage to chemical signaling. Their diversity is attributed to variations in alcohol and acyl chains, including their length, branching, and the stereochemistry of double bonds. Traditional analysis by mass spectrometry with collisional activations (CID, HCD) offers insights into acyl chain lengths and unsaturation level. Still, it falls short in pinpointing more nuanced structural features like the position of double bonds. As a solution, this study explores the application of 213-nm ultraviolet photodissociation (UVPD) for the detailed structural analysis of wax esters. It is shown that lithium adducts provide unique fragments as a result of Norrish and Norrish-Yang reactions at the ester moieties and photoinduced cleavages of double bonds. The product ions are useful for determining chain lengths and localizing double bonds. UVPD spectra of various wax esters are presented systematically, and the effect of activation time is discussed. The applicability of tandem mass spectrometry with UVPD is demonstrated for wax esters from natural sources. The UHPLC analysis of jojoba oil proves the compatibility of MS^2^ UVPD with the chromatography time scale, and a direct infusion is used to analyze wax esters from vernix caseosa. Data shows the potential of UVPD and its combination with CID or HCD in advancing our understanding of wax ester structures.

**Graphical Abstract:**

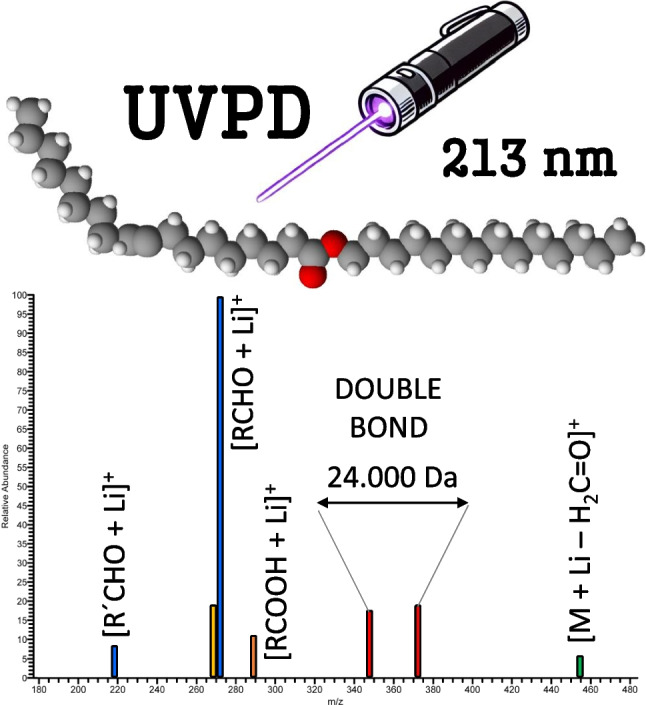

**Supplementary Information:**

The online version contains supplementary material available at 10.1007/s00216-024-05434-2.

## Introduction

Wax esters are hydrophobic lipids consisting of fatty alcohols and fatty acids linked by an ester bond. They are essential for all life, from the smallest microorganisms to the most evolved plant and animal species [1–7]. Naturally occurring wax esters are complex mixtures of many molecular species. Depending on the biosynthetic origin and function, their aliphatic chains can be straight or branched, with different lengths and numbers of double bonds [8, 9].

Wax esters have multiple biological functions, such as surface protection, energy storage, chemical communication, and sound transmission [10–15]. In mammals, wax esters are part of the sebum secreted by oil glands into hair follicles. Sebum protects the skin and helps to maintain its moisture level [5, 16]. Specialized sebaceous glands in the eyelids, Meibomian glands, produce protective lipids coating tear film on the ocular surface [9, 17]. The sebaceous glands in the immature skin of a developing human fetus also produce wax esters. They are found in vernix caseosa, a cheesy, white biofilm protecting the fetus during the last trimester of gestation. Vernix caseosa has many functions; it protects the developing skin from excessive maceration in the amniotic fluid, participates in a complex innate immune system, and helps complete intestinal development. During the transition from an intrauterine to an extrauterine environment, vernix caseosa lubricates the birth canal, helps to prevent water loss through the skin, and regulates body temperature [6, 18]. Wax esters are a common part of many foods, including cereals, leaves, seeds, and marine products. Dietary wax esters are an important source of long-chain and essential ω-3 fatty acids [19]. Insects, particularly terrestrial ones, produce wax esters as part of their cuticular lipids. These lipids form a hydrophobic layer on the insect’s exoskeleton, providing waterproofing and preventing desiccation. This protective layer also helps to deter parasites, pathogens, and harmful microorganisms from adhering to the insect’s body. Wax esters also play essential roles in intra- and interspecific communications of insects [15, 20]. Bees use wax esters to build their nests [21]. Marine crustaceans and dinoflagellates store wax esters in their bodies to provide buoyancy [7]. In plants, wax esters are constituents of the epicuticular wax layer on leaves, stems, and fruits. This layer acts as a barrier, reducing water loss through transpiration and protecting the plant from environmental stresses such as UV radiation, pathogens, and pests [22].

Although wax esters are considered simple chemical molecules, their detailed structural characterization can be challenging. The analysis is complicated by the complexity of wax ester samples, low concentration of some components, and, in the case of mammalian samples, difficult chromatographic separation from cholesteryl esters by LC-based approaches [23].

Chromatographic techniques hyphenated with mass spectrometry represent a convenient way of analyzing wax esters. Gas chromatography-mass spectrometry (GC/MS) has been previously used for analyzing wax ester samples after their hydrolysis to fatty acids and alcohols [24–26]. Nowadays, high-temperature columns offer separation of intact molecules, thus making it possible to get full information about the wax ester molecular species [10, 27–30]. In the electron ionization (EI) spectra, the most abundant wax esters (RCOOR′) fragments are protonated fatty acids [RCOOH_2_]^+^. Other abundant fragments are fatty acid radical cations [RCOOH]^+•^, acylium ions [RCO]^+^, and alcohol chain–related radical cations [R′ − H]^+•^ [10]. GC/MS has limitations connected with the low volatility of high molecular weight esters and limited thermal stability of (poly)unsaturated species [27, 31].

In the liquid phase, wax esters can be ionized using electrospray ionization (ESI), atmospheric pressure chemical ionization (APCI), and atmospheric pressure photoionization (APPI) [31–36]. All these ionization techniques produce mainly molecular adducts, which can be further fragmented to obtain structural information. Direct protonation of wax esters is inefficient in ESI [37]; however, various cations can be used to ionize these molecules. For example, jojoba oil wax esters were analyzed by LC/ESI-MS with post-column addition of silver ions. Excellent ionization was achieved, but the downside was the formation of silver oxide deposits on the ion optics [38]. English ivy cuticular wax esters were characterized by ESI-MS/MS as lithium adducts [33]. Sodium also ionizes wax esters, but it appeared unsuitable for structural elucidation because of the higher stability of sodium ion adducts [34, 37]. Ammonium ions are the most popular cationization reagent for the structural elucidation of wax esters [23, 39]. Wax esters in the form of their ammonium adducts were identified and quantified in human tears using reaction monitoring scanning [40]. Ammonium ions in the mobile phase were used to investigate wax esters causing cloudiness in canola oil [41]. Reversed-phase high-performance liquid chromatography (HPLC) with APCI-MS was applied to analyze wax esters as protonated molecules in jojoba seed oil, beeswax, and human tear fluid [31, 42, 43].

Wax ester ions are often activated collisionally to achieve structural information. The type and intensity of product ions depend on several factors, including the type of molecular adduct, instrument design, and collision energy. Fragmentation behavior is greatly affected by the presence of double bonds. In the case of saturated species, collisional activation of protonated molecules and ammonium adducts yields mostly [RCOOH_2_]^+^ and [R′]^+^. The MS/MS spectra of unsaturated esters are richer in fragments, also showing [RCO]^+^, [RCOO]^+^, and various dehydration products of precursor ions and fragments, depending on the number and location of double bonds. Cleavages of aliphatic chains occur when at least one double bond is present, yielding several low-intensity peaks. However, the double bond position cannot be determined from these fragments [23, 31, 34].

Although many methods have been developed for determining the double bond positions in lipids [44], few have been applied to wax esters. In GC/EI-MS, dimethyl disulfide derivatives are useful to localize double bonds in wax esters [45]. Radical ions are involved in processes employed in APCI MS methods. Reactive C_3_H_5_N^+•^ ions formed from acetonitrile react with double bonds to form [M + C_3_H_5_N]^+•^ products [46]. Collision activation of the mass-selected product ions yields fragments unambiguously localizing the double bond position. In addition to wax esters, the method applies to many aliphatic lipids, including fatty acid methyl esters, methyl esters of hydroxy fatty acids, diol esters, triacylglycerols, and wax esters [47–53].

In recent years, we have witnessed the rapid development of new ion activation methods and their implementation in commercial mass spectrometers. With the help of new tandem mass spectrometry methods, such as electron-induced dissociation (EID) or ultraviolet photodissociation (UVPD), we can better describe lipid molecules [54, 55]. In EID, multiple interactions with electrons lead to electronic and vibrational excitations, yielding information-rich spectra with extensive cleavages across the acyl chains. For example, EID made it possible to determine the double bond position in Mn(II) adducts of fatty acid [56]. FT-ICR with EID provided information-rich spectra with specific product ions for characterizing acyl positions and double bonds in glycerophospholipids [57]. In this way, phosphatidylcholines desorbed and ionized from tissue samples by MALDI could be analyzed [58].

In UVPD, a laser beam of high-energy photons excites ions to higher electronic states [59]. Higher energy decomposition pathways are accessed, resulting in fragments not achievable by low-energy collisional activation. Fragmentation occurs due to photon absorption to various moieties of the analyte, such as double bonds [60–62]. The utility of UVPD for the structural characterization of lipids has been demonstrated in several studies. UVPD was used to localize double bonds in phosphatidylcholines and sphingolipids, with the charge fixed on their polar head groups [63, 64]. Later, it was shown that sodium adducts allow for the determination of double bond position in a range of glycerolipids [65]. Photoionization of deprotonated glycerophospholipids made it possible to identify the headgroup, acyl chain position, and double bond position [66]. UVPD proved useful for characterizing cyclopropane rings in bacterial phospholipids [67]. Another study demonstrated the ability of UVPD to provide detailed structural information on lithiated sterols [68]. Fragmentation of lithium adducts of fatty acids by UVPD at 193 nm provided ions specific for the position of double bonds [69]. In fatty acid esters of hydroxy fatty acids (FAHFA), UVPD of [M + Li]^+^ and [M – H + 2Li]^+^ made it possible to identify the position of ester moiety and double bonds [70].

This paper explores the utility of UVPD at 213 nm for the structural characterization of wax esters. It is demonstrated that UVPD makes it possible to determine the length of acid and alcohol chains and localize double bonds. The UVPD spectra of lithium adducts suggest that photochemical Norrish and Norrish-Yang reactions are involved. A pair of 1,2-elimination products differing by 24.0000 Da provides unambiguous localization of a double bond. UHPLC/MS^2^ UVPD method for wax ester from jojoba oil was developed, and the main wax esters were identified. Abundant wax esters in vernix caseosa were annotated using data-independent MS^2^ UVPD and MS^3^ CID/UVPD workflows.

## Materials and methods

### Solvents and reagents

Acetonitrile (LC-MS LiChrosolv^®^), toluene (Chromasolv^®^ Plus, for HPLC), lithium formate monohydrate (≥ 98%), potassium acetate (ASC reagent grade), and ammonium acetate (LC-MS LiChropur) were purchased from Sigma-Aldrich (St. Louis, MO, USA). Sodium formate (for HPLC), 2-propanol (LC-MS Chromasolv^®^), and chloroform (for HPLC ≥ 99.8%) were from Honeywell/Riedel-de Haën (Seelze, Germany).

### Wax ester standards and samples

Wax ester standards (Table S1 in Supporting Information (SI)) purchased from Nu-Chek Prep (Elysian, MN, USA) were dissolved in chloroform (1.0 mg/ml) and further diluted with 2-propanol:methanol:chloroform (4:2:1, by vol.) containing 2.0 mmol/l lithium formate to the concentration of 10.0 µmol/l. Lithium formate was replaced with sodium formate, potassium acetate, or ammonium acetate for experiments with other types of cationization. The sample solutions (20.0 µl) were loaded into the wells of the Eppendorf twin-tec 96-well PCR plate.

### Wax esters isolated from natural samples

A weight of 15.0 mg of jojoba oil (Jojoba oil bio, Primavera Life, Sulzberg, Germany) was dissolved in 100 µl of chloroform. Vernix caseosa was collected from the back of a healthy newborn boy delivered in 39 gestation weeks. The sample was collected with written informed parental consent, and the study was approved by the Ethics Committee of the General University Hospital in Prague, Czech Republic (910/09 S-IV). The collected vernix caseosa sample (531 mg) was suspended in 50 ml of chloroform:methanol mixture (2:1, by vol.) with 0.05% of butylated hydroxytoluene. The extract was dried over the anhydrous MgSO_4_ and filtrated through a precleaned cotton wool and silica gel (60–120 µm, ca 0.2 g).

The lipid extracts were separated on in-house-made precleaned glass TLC plates (9 × 12 cm) coated with silica gel 60 G for thin-layer chromatography (Merck; Darmstadt, Germany) using hexane:diethyl ether (93:7, by vol.) mobile phase. The lipid extract was developed twice to focus the zones (in the first step to 3/4 of the plate height and then, after air drying, to the top). TLC zones were visualized by spraying with Rhodamine 6G solution (0.05% in ethanol) and inspecting the plates under UV light (254 nm). The zone of wax esters (identified by TLC of wax ester standards, R_f_ 0.65–0.75) was scratched off the plate into a glass column with purified cotton wool and ca 100 mg of silica gel at the bottom. Wax esters were eluted with freshly distilled diethyl ether. The solvent was removed by a nitrogen stream [71]. Jojoba oil wax esters were dissolved in methanol:chloroform (1:1, by vol.) to a concentration of 0.5 mg/ml. Vernix caseosa wax esters reconstituted in chloroform (10 mg/ml) were diluted with 2-propanol:methanol:chloroform (4:2:1, by vol.) containing 2.0 mmol/l lithium formate to a concentration of 1 mg/ml.

### Direct infusion mass spectrometry

Mass spectra of standards and wax esters from vernix caseosa were collected with the Orbitrap Fusion Lumos Tribrid (Thermo Fisher Scientific, San Jose, CA) equipped with a 213 nm CryLaS laser system with a 2.5 kHz repetition rate delivering > 1.2 µJ per pulse. The samples were delivered using an Advion Triversa Nanomate ionization source (Advion Inc., Ithaca, NY) operated with a spray voltage of 1.4 kV and a gas pressure of 0.3 psi. The inlet capillary temperature was set to 275 °C. The target *m/z* ± 0.5 Da as the isolation window for precursor selection was used. The automatic gain control target of the Orbitrap mass analyzer was maintained at 800%, and the maximum injection time was 100 ms. UVPD spectra of wax ester standards were collected using an activation time of 500 ms. UVPD spectra of wax esters from jojoba oil and vernix caseosa were obtained with activation times of 700 ms and 2000 ms, respectively. MS^2^ HCD and CID spectra were typically acquired with a normalized collision energy of 40% and 30%, respectively. Orbitrap mass resolution was set at 120,000 (FWHM; specified for *m/z* 200). Data were evaluated manually based on elemental composition predicted by the Xcalibur Qual Browser (Thermo) software.

### High-performance liquid chromatography

The UHPLC/MS^2^ experiments were performed with Dionex Ultimate 3000 LC system (Thermo Scientific, San Jose) interfaced with the Orbitrap Fusion Lumos Tribrid mass spectrometer equipped with a heated ESI source. The samples (solution of wax esters from jojoba oil) were injected into a Waters Acquity BEH C18 column (2.1 × 50 mm, 1.7 µm particle size) kept at 45 °C. The separation was achieved in 40 min using a mobile phase gradient programmed from phase A (water:acetonitrile 4:6, with 10 mM ammonium acetate and 0,1% formic acid) and B (acetonitrile:2-propanol 1:9, with 10 mM ammonium acetate and 0.1% formic acid) as follows: 0 min - 30% B; 5.0 min - 70% B; 35.0 min - 100% B; 40.0 min - 30% B; the mobile phase flow rate was 180 µl/min. Lithium formate solution (0.5 mmol/l in 2-propanol:methanol:chloroform 4:2:1) was infused post-column at 10 µl/min flow rate using a tee-union. The heated ESI source was set as follows: spray voltage of 3.5 kV, sheath gas of 40 a.u., auxiliary gas of 3 a.u., sweep gas of 2 a.u., ion transfer tube temperature of 320 °C, and vaporizer temperature of 300 °C. The MS/MS spectra in data-dependent experiments were acquired at a resolving power of 15,000 with an isolation window of 1.2 Da. The maximum injection time was 100 ms.

### Nomenclature of wax esters

Shorthand notations of wax ester structure follow the format used by LIPID MAPS [72, 73]. The first part of wax ester abbreviations refers to the alcohol segment of the molecule, whereas the second part indicates the fatty acid. The general formula of wax esters is RCOOR′, where R and R′ are the alkyl moieties of the acid and alcohol part, respectively.

## Results and discussion

Mass spectra of collisionally activated wax ester ions are well documented in the literature [31, 35, 74]. In this work, we systematically studied UVPD spectra of saturated, monounsaturated, and polyunsaturated wax esters to determine fragmentation upon exposure to high-energy photons.

As various adducts may exhibit distinct fragmentation pathways due to different binding sites and energies involved [23, 46], we investigated the effect of the type of molecular adduct on MS^2^ UVPD spectra. The experiments described in Supplementary Information (Text S1, Figures S1, S2, and S3) showed that lithiated wax esters provided more informative spectra than Na^+^, K^+^, NH_4_^+^, and H^+^ adducts. In addition to the adduct type, the laser activation time is an important parameter in UVPD. In the Orbitrap Fusion Lumos, it is the time that the pulsed laser beam irradiates the mass-selected ions in the ion trap. Increasing the activation time typically results in more extensive fragmentation of the ions. We were interested in the lowest activation time that provides quality spectra and the effect of the activation time on the relative abundances of fragment ions. Therefore, MS^2^ UVPD spectra of lithiated WE(16:0/14:1(9Z)) and WE(12:0/18:2(9Z,12Z) were recorded using 10 to 4000 ms activation times (Text S2 and Figures S4–S9 in SI). The abundance of the fragments increased with increasing activation time. The minimum activation time required to obtain spectra with all expected fragments was 100 ms; however, longer activation times were required to ensure sufficient reproducibility. For directly infused standards (1.0 mg/ml), the activation time of 500 ms was selected to get good fragment intensities and an acceptable scan time. No significant changes in the relative abundances of the fragments were noticed at even very long activation times. Longer activation times improve spectra quality; however, in practical applications, the activation time must also reflect the required scan speed.

### UVPD spectra of saturated wax ester standards

**Fig. 1:**
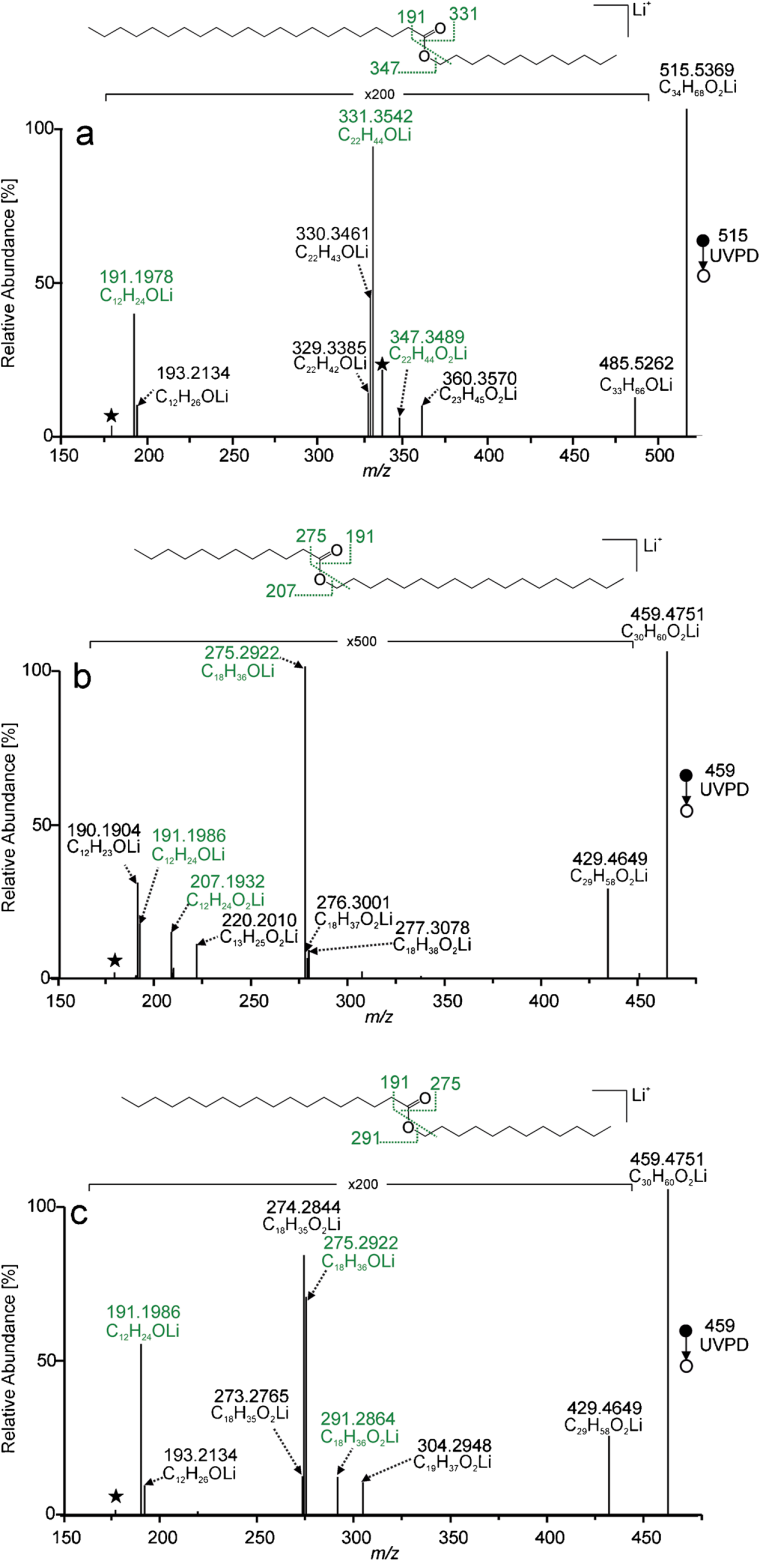
MS^2^ UVPD spectra of [M + Li]^+^ of lauryl behenate (WE(12:0/22:0)) (**a**), stearyl laurate (WE(18:0/12:0)) (**b**), and lauryl stearate (WE(12:0/18:0)) (**c**) recorded using the activation time of 500 ms. Lithiated aldehyde and fatty acid fragments are highlighted in green; the star (⋆) indicates an FT artifact. Cleavages indicated in the structural formulas respect the proposed fragmentation mechanism

**Scheme 1:**
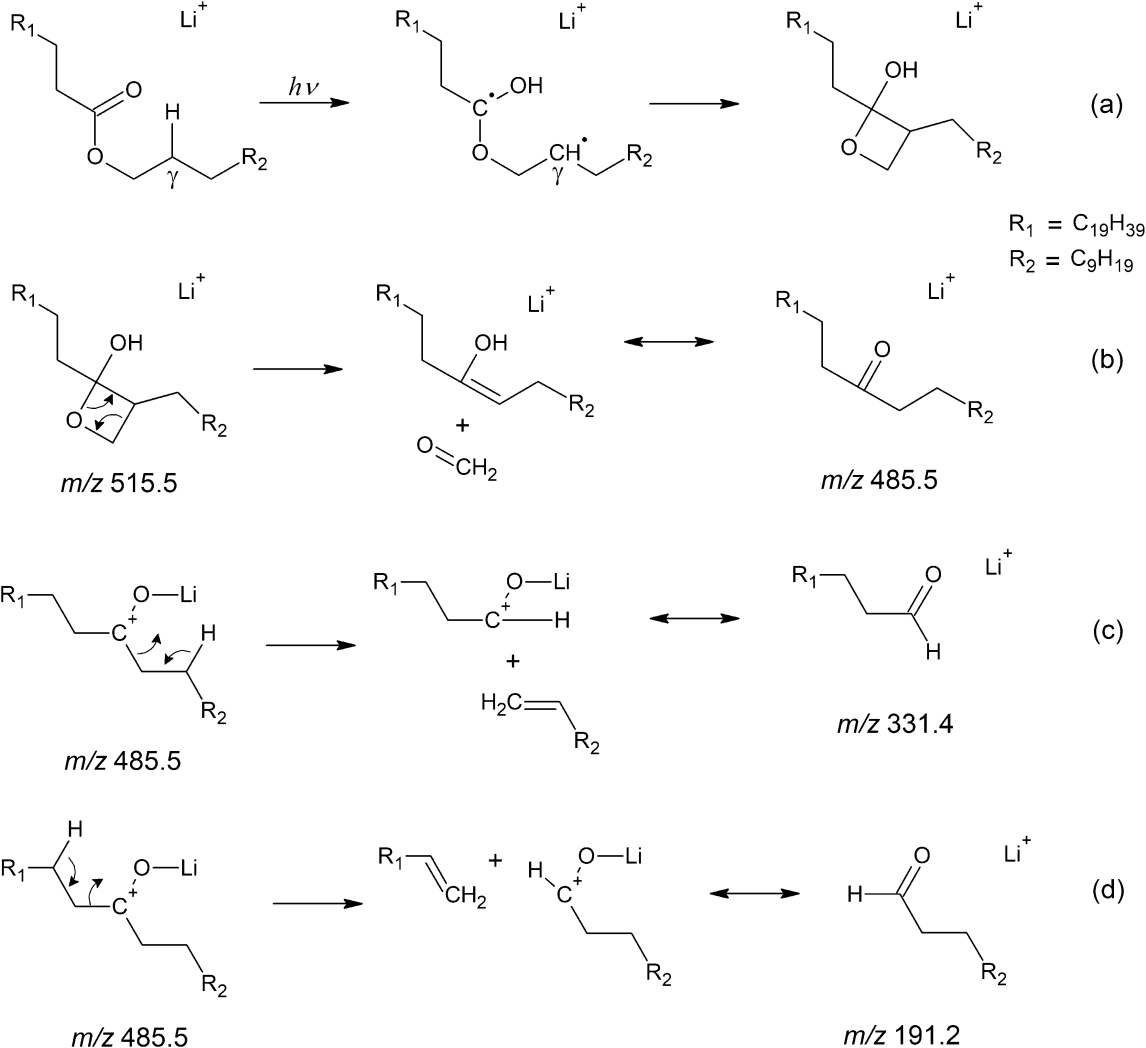
Proposed UVPD fragmentation pathways: Norrish-Yang photochemical cyclization of WE(12:0/22:0) (**a**), cleavage of the disubstituted oxetan-2-ol by neutral loss of formaldehyde (**b**), and cleavages of the lithiated ketone leading to lithiated aldehydes related to the acid (**c**) and alcohol (**d**) chains of the wax ester

Saturated wax esters were investigated first. MS^2^ UVPD spectrum of lithiated lauryl behenate WE(12:0/22:0) is shown in Fig. 1a. The main fragment *m/z* 331.3542 (C_22_H_44_OLi^+^) bearing information on the acyl chain length was interpreted as lithiated docosanal. The alcohol part of the wax ester manifested itself by another aldehyde ion *m/z* 191.1978 (C_12_H_24_OLi^+^; lithiated dodecanal). The spectrum also showed a fragment *m/z* 485.5262 (C_33_H_66_OLi^+^) consistent with eliminating formaldehyde from the lithiated wax ester precursor. We suggest that these fragments in the UVPD spectra could be due to reactions initiated by a photochemical Norrish-Yang cyclization [75, 76] leading to disubstituted oxetan-2-ol (Scheme 1a). Cleavage of the ring liberated formaldehyde upon forming an enol, which tautomerized to tritriacontane-12-one (Scheme 1b). Its lithium adduct fragmented to lithiated docosanal and dodecanal after eliminating corresponding 1-alkenes (Scheme 1c, d). Alternatively, lithiated aldehydes could be formed directly from the disubstituted oxetan-2-ol. Assigning the aldehyde ions to the acid and alcohol part in a spectrum of an unknown wax ester would not be difficult. The aldehyde ion, which came from the acid part of the ester, was accompanied by two ions, one and two hydrogens smaller. In the case of WE(12:0/22:0), these fragments were *m/z* 330.3461 (C_22_H_43_OLi^+^) and *m/z* 329.3385 (C_22_H_42_OLi^+^). The former could be explained by a Norrish type I reaction, i.e., homolysis of the ester bond into two radical intermediates: acyl and alkoxyl radicals (Scheme S-Ia in SI) [77]. Lithiated acyl radical (*m/z* 330.3461) was stabilized by eliminating hydrogen in the α-position upon forming lithiated ketene (*m/z* 329.3385) (Scheme S-Ib in SI). The acid part of the ester was also identified by the ion *m/z* 347.3489 (C_22_H_44_O_2_Li^+^), which corresponded to the lithiated behenic acid. This ion could be explained by Norrish type II [78] reaction or an alternative cleavage of the four-membered oxetan-2-ol ring, in this case, accompanied by the neutral loss of 1-dodecene (Scheme S-II in SI). The spectrum also revealed an acid-related radical fragment *m/z* 360.3570 (C_23_H_45_O_2_Li^+•^). The structure of this fragment, having one more carbon atom than the acid part of the wax ester, is unclear, as is its formation mechanism. The aldehyde ion corresponding to the alcohol chain of the wax ester was not accompanied by fragments one and two mass units smaller, like in the case of acid chain-related aldehyde fragment. The alcohol part was also characterized by *m/z* 193.2134 (C_12_H_26_OLi^+^), lithiated dodecanol, likely formed by Norrish type I reaction followed by hydrogen transfer as shown in Scheme S-Ib in SI. The activation of WE(12:0/22:0) by UV photons provided a different set of fragments than collisional activations. MS^2^ CID (Figure S10a in SI) and MS^2^ HCD (Figure S10b in SI) spectra were much simpler, with lithiated behenic acid (*m/z* 347.3494; C_22_H_44_O_2_Li^+^) as the sole fragment.

Saturated esters stearyl laurate (WE(18:0/12:0)) and lauryl stearate (WE(12:0/18:0)) are isomers differing by the ester group position. They can be distinguished according to their acid fragments in CID spectra (Figure S11 in SI). The MS^2^ UVPD spectra (Fig. 1b, c) also made distinguishing between these isomers possible. A key step was determining which aldehyde ions corresponded to the acid and alcohol portions of the esters. For these two esters, the aldehyde ions were found at *m/z* 191.1986 (C_12_H_24_LiO^+^) and *m/z* 275.2922 (C_18_H_36_LiO^+^). The aldehyde peak from the alcohol portion was accompanied by an alcohol peak at nominal *m/z* greater by two. Such peaks were detected at *m/z* 277.3078 (C_18_H_38_LiO^+^) for WE(18:0/12:0) and *m/z* 193.2134 (C_12_H_26_LiO^+^) for WE(12:0/18:0). The aldehyde peak from the acid portion of the ester was accompanied by an acyl radical peak at one nominal mass unit lower. In our example, these peaks were found at *m/z* 190.1904 (C_12_H_23_LiO^+^) for WE(18:0/12:0) and *m/z* 274.2844 (C_18_H_35_LiO^+^) for WE(12:0/18:0). Identification of aldehyde ions based on their accompanying peaks was reliable and led to unambiguous structure assignment. If the wax ester had the same number of carbons in both the acid and alcohol parts, only one aldehyde ion was formed. Fragments of both types then accompanied it, the alcohol ion at nominal *m/z* + 2 and the acyl radical at nominal *m/z* − 1 (see the spectrum of WE(14:0/14:0) in Figure S12 in SI).

### UVPD spectra of unsaturated wax ester standards

**Fig. 2:**
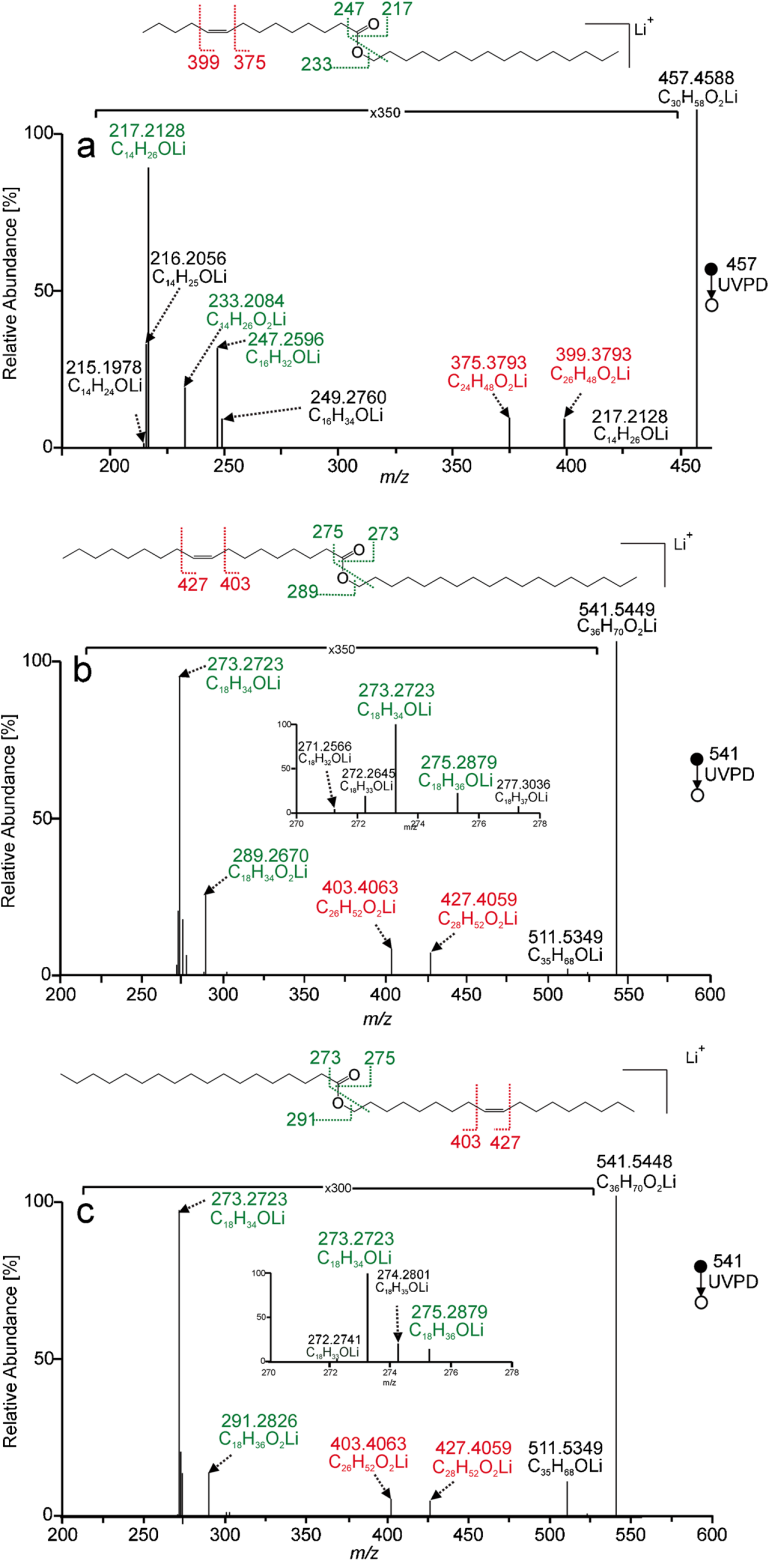
MS^2^ UVPD spectra of [M + Li]^+^ of palmityl myristoleate (WE(16:0/14:1(9Z))) (**a**), stearyl oleate (WE(18:0/18:1(9Z))) (**b**), and oleyl stearate (WE(18:1(9Z)/18:0)) (**c**) recorded using the activation time of 500 ms. Lithiated aldehyde and fatty acid fragments are highlighted in green, and the pair of fragments indicating the position of the double bond is marked in red

The advantages of UVPD for structural analysis were particularly evident in the case of wax esters with unsaturated aliphatic chains. Unlike collisional activations, UVPD spectra made it possible to determine the position of double bonds. In the spectrum of WE(16:0/14:1(9Z), Fig. 2a, all types of fragments discussed above were detected. Major aldehyde peak *m/z* 217.2128 (C_14_H_26_LiO^+^) accompanied by *m/z* 216.2056 (C_14_H_25_LiO^+^) and *m/z* 215.1978 (C_14_H_24_LiO^+^) characterized fatty acid chain, whereas less intense aldehyde ion *m/z* 247.2596 (C_16_H_32_LiO^+^) accompanied by *m/z* 249.2760 (C_16_H_34_LiO^+^) described the alcohol moiety. Lithiated myristoleic acid *m/z* 233.2084 further confirmed the acid moiety. Besides these ions, two fragments separated by the mass of two carbon atoms (24.0000 Da) at *m/z* 399.3793 (C_26_H_48_LiO_2_^+^) and *m/z* 375.3793 (C_24_H_48_LiO_2_^+^) were detected. They were formed by 1,2-elimination after the absorption of UV photons into the double bond. The reaction (Scheme S-III in SI) described earlier for phosphatidylcholines [64] and lithiated sphingolipids [63] made localizing the double bond position possible. The location of the double bond on the acid or alcohol chain could be determined from the degree of unsaturation of the corresponding aldehyde ions. In this case, the fragments indicated the n − 5 double bond in the acid chain. CID and HCD spectra of unsaturated wax esters did not allow the double bond position to be determined. The MS^2^ CID and MS^2^ HCD of WE(16:0/14:1(9Z)) provided only one fragment, lithiated myristoleic acid (Figure S13 in SI). Identification of aldehyde ions in MS^2^ UVPD spectra of monounsaturated wax esters might not be straightforward when both chains have the same number of carbon atoms. In such cases, nominal *m/z* – 2 and *m/z* + 2 accompanying peaks can be at the same masses as the aldehyde ions. For instance, the region around *m/z* 273.2723 in WE(18:0/18:1(9Z)) and WE(18:1(9Z)/18:0) spectra looked similar (Fig. 2b, c). However, the n − 9 double bond manifested by *m/z* 403.4063 and *m/z* 427.4059 could be unambiguously assigned to the acid or alcohol chain based on lithiated fatty acid ions, *m/z* 289.2670 and *m/z* 291.2826, respectively.

**Fig. 3:**
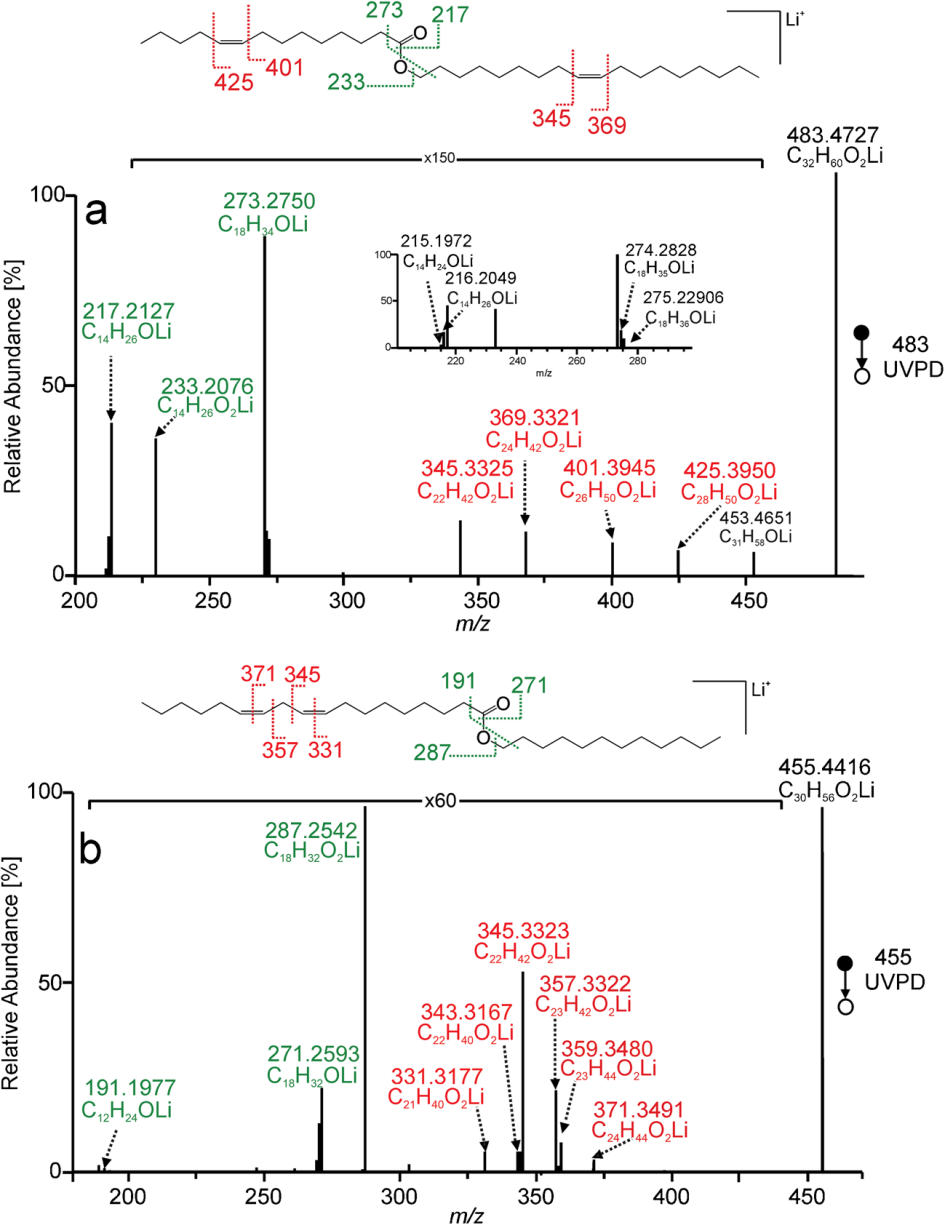
MS^2^ UVPD spectra of [M + Li]^+^ of oleyl myristoleate (WE(18:1(9Z)/14:1(9Z))) (**a**) and lauryl linoleate (WE(12:0/18:2(9Z,12Z))) (**b**) recorded using the activation time of 500 ms. Lithiated aldehyde and fatty acid fragments are highlighted in green, and the fragments indicating the position of double bonds are marked in red

Diunsaturated wax esters can have one double bond in each chain or both double bonds in the same chain. In the spectrum of WE(18:1(9Z)/14:1(9Z)) in Fig. 3a, aldehyde ions were easily assigned based on accompanying peaks; the acid chain-related aldehyde at *m/z* 217.2127 (C_14_H_26_OLi^+^) and the alcohol chain-related aldehyde at *m/z* 273.2750 (C_18_H_34_OLi^+^). Two pairs of fragments indicating double bond were present: n − 5 (*m/z* 401.3945 and *m/z* 425.3950) and n − 9 (*m/z* 345.3325 and *m/z* 369.3321). The MS^2^ UVPD spectrum did not allow us to assign double bonds to acid and alcohol chains. However, the problem could be resolved by the MS^3^ CID/UVPD experiment. Lithiated fatty acid (*m/z* 233.2082, C_14_H_26_O_2_Li^+^) generated in CID provided the n − 5 diagnostic fragments (*m/z* 151.1300 and *m/z* 175.1299) in UVPD (Figure S14 in SI). Therefore, the n − 5 double bond was in the acid chain, and the n − 9 had to be in the alcohol chain. UVPD in the MS^3^ step was limited by the lower intensity of the precursor ion; a higher activation time (2000 ms) was required to obtain the diagnostic fragments at sufficient intensities.

In the case of both double bonds in the same chain, like in WE(12:0/18:2(9Z,12Z)) (Fig. 3b), the identification of the acid and alcohol chains was straightforward. The acid part could be determined by the aldehyde ion at *m/z* 271.2593 (C_18_H_32_OLi^+^) and the strong signal of lithiated 18:2 acid at *m/z* 287.2542 (C_18_H_32_O_2_Li^+^). The two double bonds separated by one methylene group were manifested by several peaks in the *m/z* 331–371 range. They corresponded to cleavages of all C=C and C−C bonds in the methylene-interrupted diene moiety. The fragmentation pattern was almost identical also for other wax ester standards with n − 6 methylene interrupted double bonds (Figure S15 in SI). The most abundant signals in this range, *m/z* 345.3323 and *m/z* 357.3322, corresponded to C10−C11 and C11−C12 bond cleavages, i.e., single bonds nested between two double bonds. These signals were separated by 12 Da, which can be a useful diagnostic feature for localizing methylene-interrupted double bonds.

**Fig. 4:**
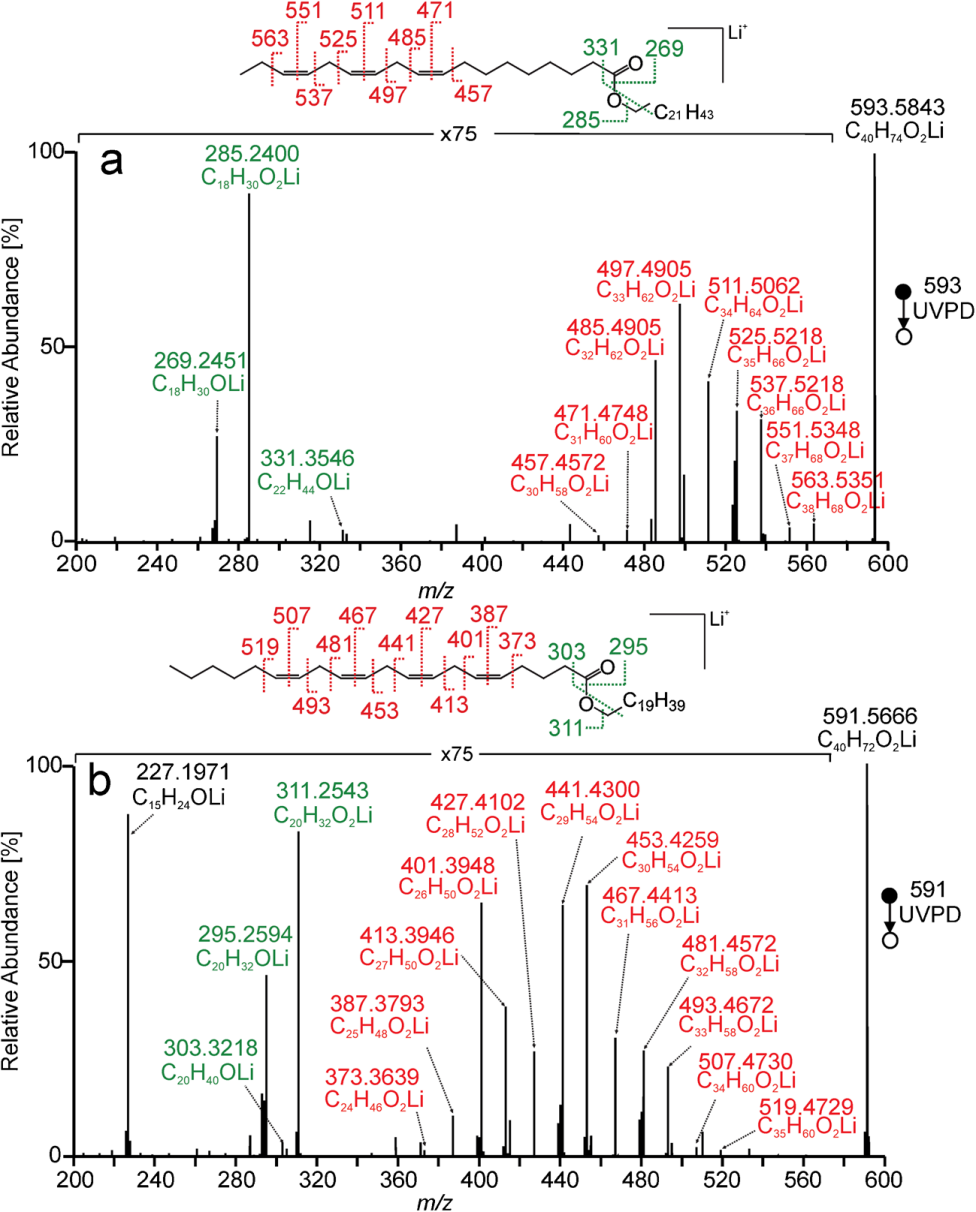
MS^2^ UVPD spectrum of [M + Li]^+^ for behenyl α-linoleate (WE(22:0/18:3(9Z,12Z,15Z))) (**a**), arachidyl arachidonate (WE(20:0/20:4(5Z, 8Z,11Z,14Z))) (**b**), recorded using the activation time of 500 ms. Lithiated aldehyde and fatty acid fragments are highlighted in green, and the fragments indicating double bond positions are marked in red

Wax ester WE(22:0/18:3(9Z,12Z,15Z)) showed lithiated fatty acid at *m/z* 285.2400 (C_18_H_30_O_2_Li)^+^ as the most abundant fragment (Fig. 4). The acid chain-related aldehyde *m/z* 269.2451 (C_18_H_30_OLi)^+^ was much more intense than alcohol chain-related aldehyde *m/z* 331.3546 (C_22_H_44_OLi)^+^. Abundant fragments in the *m/z* 457–563 range indicated three methylene-interrupted double bonds in the n − 3 position. Determining the double bond positions was not entirely straightforward. As in the case of wax esters with two methylene-interrupted double bonds, fragments corresponding to cleavages of C–C bonds linking the methylene group to carbons of the adjacent double bonds gave intense signals separated by 12 Da (for C11 *m/z* 485.4905 and *m/z* 497.4905, for C14 *m/z* 525.5218 and *m/z* 537.5218). Less intense signals delimiting the entire region of double bond fragments could also be used to determine the position of the methylene-interrupted double bond system. However, the practical use of these ions is limited by their low intensity. The spectra became even more complicated if several double bonds were distributed in both wax ester chains. Therefore, comparing entire spectra or spectra regions with double bond-related fragments to standards is worthwhile. In the spectra of arachidonic acid esters, an abundant fragment *m/z* 227.1971 (C_15_H_24_OLi)^+^ was present (Fig. 4b, Figure S16 in SI). We hypothesize that the reaction leading to this fragment involves the first double bond on C5 of the arachidonic acid chain.

### UHPLC/ESI-MS^2^ UVPD of wax esters in jojoba oil

The applicability of UVPD for characterizing wax esters separated by liquid chromatography was investigated using esters isolated from jojoba oil [31, 38, 42, 46]. The UHPLC was achieved with the BEH C18 column and a mobile phase consisting of 2-propanol, water, and acetonitrile with ammonium acetate and formic acid. The post-column addition of lithium formate ensured the formation of lithium adducts.

**Fig. 5:**
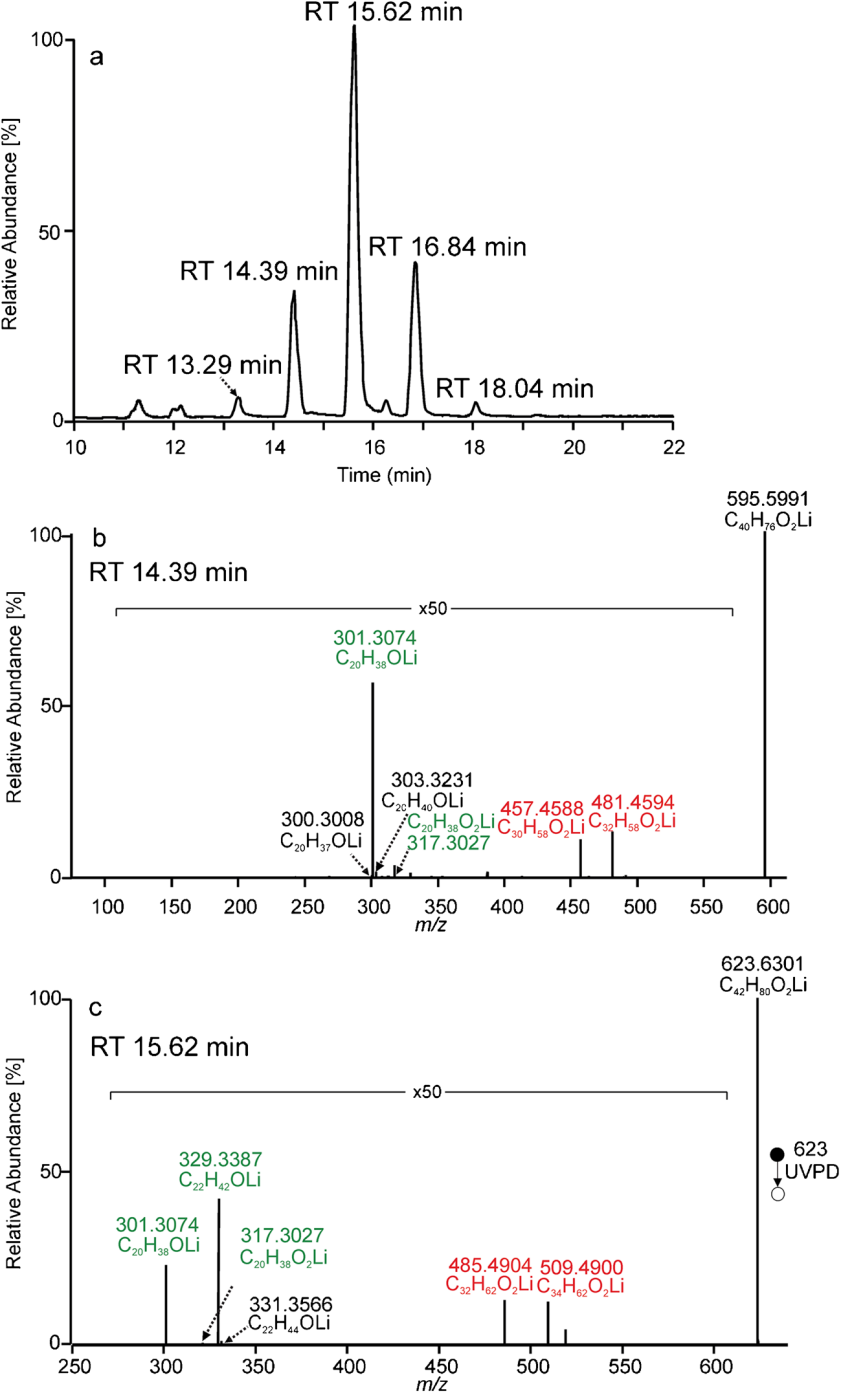
Chromatogram of jojoba oil wax esters (**a**), MS^2^ UVPD spectrum of the peak at RT 14.39 min identified as WE(20:1(11)/20:1(11)) (**b**), and MS^2^ UVPD spectrum of the peak at RT 15.62 min identified as WE(22:1(13)/20:1(11)) (**c**) recorded using the activation time of 500 ms. Lithiated aldehyde and fatty acid fragments are highlighted in green, and the fragments indicating the position of the double bond are marked in red

Wax esters were detected by data-dependent analysis involving MS^1^ full scan in Orbitrap followed by MS^2^ UVPD of precursor ions selected from an inclusion list. The MS^2^ UVPD was performed using the activation times of 100 ms, 500 ms, and 700 ms. The shortest activation, 100 ms, provided spectra with too low intensities of diagnostic fragments. Major signals were present, but minor peaks confirming acid and alcohol-related aldehyde fragments and double bond–related fragments were mostly absent or indistinguishable from noise. Good-quality spectra were obtained using activation times of 500 ms and 700 ms (Figure S17 in SI). The results discussed further relate to data taken at the activation time of 700 ms, which delivered slightly higher intensities of fragments. The base peak chromatogram of wax esters ([M + Li]^+^) from jojoba oil is shown in Fig. 5a, and the identified species are presented in Table 1. Figure 5b and c show MS^2^ UVPD spectra taken across chromatographic peaks eluting in 14.39 min and 15.62 min, respectively. The first spectrum was consistent with WE(20:1(11)/20:1(11)). The mass of the precursor ion *m/z* 595.5991 (C_40_H_76_O_2_Li^+^) indicated an ester with 40 carbons and two double bonds. One aldehyde fragment *m/z* 301.3074 (C_20_H_38_OLi^+^) accompanied by minor signals at *m/z* 303.3231 and *m/z* 300.3008 showed that both aliphatic chains had the same length. The acid chain length was further confirmed by lithiated C20:1 fatty acid at *m/z* 317.3027. Since only one pair of fragments indicating the n − 9 double bond was present (*m/z* 457.4588, *m/z* 481.4594), the same position of double bonds in both chains was concluded. The second MS^2^ UVPD spectrum (Fig. 5c) was generated from *m/z* 623.6301. The precursor mass revealed 42 carbons and two double bonds. Two aldehyde fragment ions existed in the spectrum: *m/z* 301.3074 (C_20_H_38_OLi^+^) and *m/z* 329.3387 (C_22_H_42_OLi^+^). The first fragment was accompanied by a nominal *m/z* + 2 ion at *m/z* 331.3566 (C_22_H_44_OLi^+^), suggesting the alcohol chain. The second aldehyde fragment did not show any nominal *m/z* + 2 peak. Instead, a nominal *m/z* + 16 peak was detected at *m/z* 317.3027 (C_20_H_38_O_2_Li^+^), indicating C20:1 acid. The *m/z* 485.4904/*m/z* 509.4900 pair bore information about n − 9 double bond. Therefore, the spectrum was interpreted as WE(22:1(13)/20:1(11)). Other abundant wax esters in jojoba oil were identified similarly. The results shown in Table 1 agreed with the published data [31].

**Table 1:** Wax esters found in jojoba oil and fragments used for their identification

RT (min)	Precursor ion	Acid-related aldehyde	Alcohol-related aldehyde	Acid fragment	Double bond-related fragments	Identification
13.29	567.5681	273.2760	301.3074	289.2722	429.4280, 453.4280	20:1(n − 9)/18:1(n − 9)
14.39	595.5996	301.3074	301.3074	317.3090	457.4588, 481.4594	20:1(n − 9)/20:1(n − 9)
15.62	623.6305	301.3074	329.3389	317.3027	485.4902, 509.4202	22:1(n − 9)/20:1(n − 9)
16.84	651.6624	301.3077	357.3703	-	513.5220, 537.5222	24:1(n − 9)/20:1(n − 9)
18.06	679.6903	357.3706	329.3387	-	541.5561, 565.5535	22:1(n − 9)/24:1(n − 9)

### ESI-MS^2^ UVPD and ESI-MS^3^ CID/UVPD shotgun analysis of wax esters in vernix caseosa

Shotgun analysis of lipid samples offers a simple and high-throughput approach suited for large-scale studies. It provides virtually unlimited time for fragmentation experiments with each precursor, which can be an advantage over chromatographic methods. We tested MS^2^ UVPD shotgun analysis on a complex mixture of wax esters from vernix caseosa. The sample was infused into the ESI source, and the most abundant lithiated precursors were manually selected and fragmented by UVPD with an activation time of 2000 ms.

**Fig. 6:**
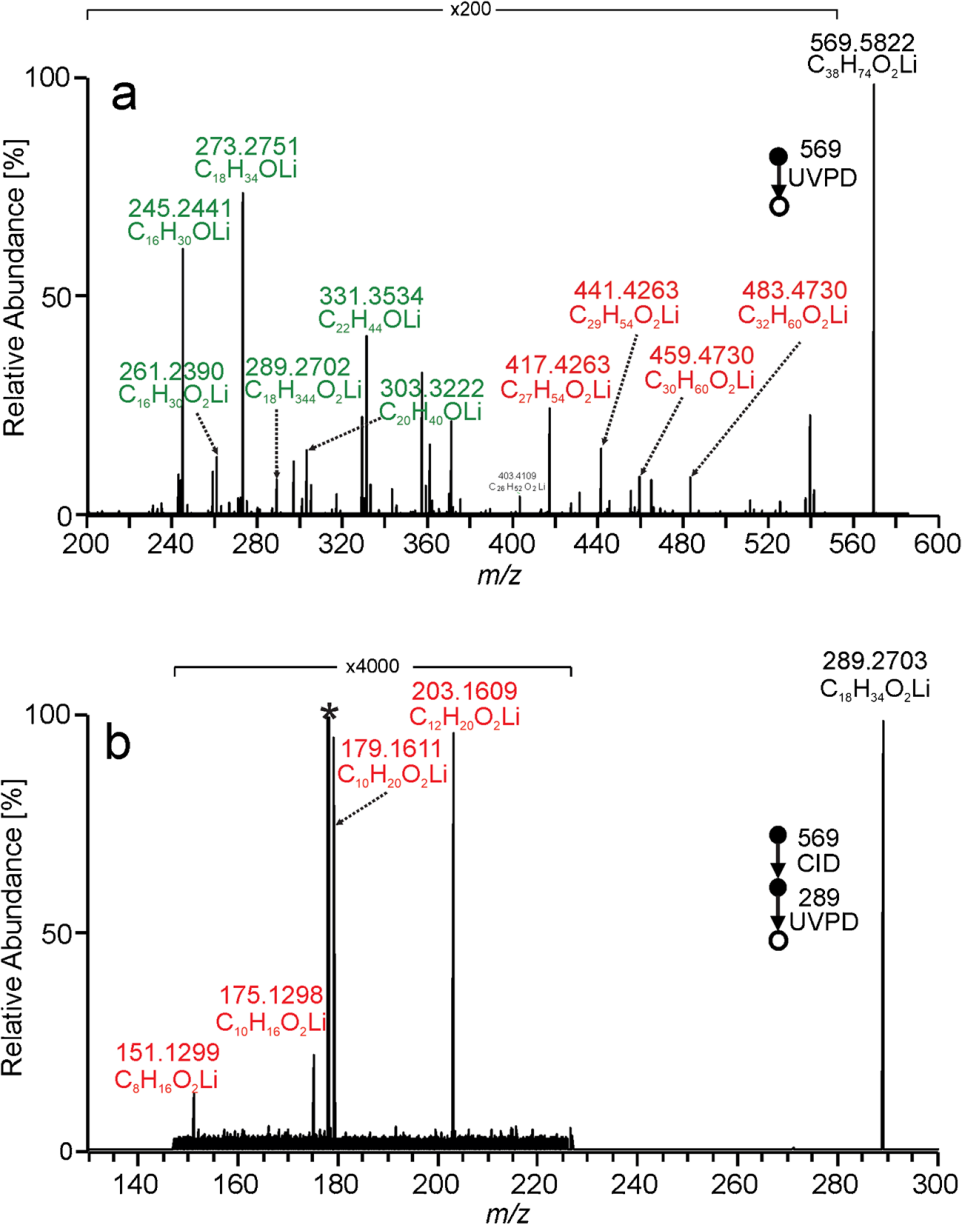
MS^2^ UVPD spectrum of *m/z* 569.5822 (C_38_H_74_O_2_Li) from vernix caseosa (**a**) showing lithiated aldehyde and fatty acid fragments highlighted in green and the fragments indicating the position of double bond marked in red. MS^3^ CID/UVPD of *m/z* 569.6 / *m/z* 289.3 from vernix caseosa (**b**) showing pairs of diagnostic fragments for double bonds in n − 9 and n − 7 positions

Lithium adducts of wax esters were observed in the *m/z* 400–650 range (Figure S18 in SI); signals at higher *m/z* values corresponded to cholesteryl esters. The interpretation procedure is explained for *m/z* 569.5822, i.e., wax ester with 38 carbons and one double bond (Fig. 6). In the first step, acid and alcohol chain-related aldehyde fragments, e.g., ions containing one oxygen, were identified. For each of these ions, we searched for − 2H, − H, and + O fragments that suggested acid-related aldehyde fragments and + 2H signals pointing to alcohol-related fragments. This way, 16:1 and 18:1 fatty acyls and 20:0 and 22:0 alcohol chains were found, giving WE(20:0/18:1) and WE(22:0/16:1) as major species. Wax esters WE(16:1/22:0), WE(24:0/14:1), WE(18:1/20:0), and WE(16:1/22:0) were identified as less abundant or minor components. In the next step, we focused on fragments containing two oxygen atoms. They were either lithiated fatty acids consistent with acid chain-related aldehyde fragments identified in the previous step or fragments indicating the double bond position. Among these ions, we searched for pairs of fragments 24.0000 Da apart. We also checked whether the fragment intensities within each pair were similar. The fragment pairs with the highest intensities, *m/z* 417.4263/441.4263 and *m/z* 459.4730/483.4730, indicated n − 10 and n − 7 double bond positions, respectively. Less abundant fragments revealed n − 13, n − 12, n − 11, n − 9, and n − 5 double bonds.

An MS^3^ CID/UVPD experiment was used to get more detailed information about the structure of wax esters in vernix caseosa. Wax ester precursors were fragmented by CID to generate lithiated fatty acids. These fragments were mass-selected in the ion trap and subjected to UVPD with an activation time of 2000 ms. This way, information about double bond positions in fatty acid chains was obtained. For instance, MS^3^ CID/UVPD spectrum (*m/z* 569.6/*m/z* 289.3) displayed diagnostic pairs *m/z* 151.1299/175.1299 (n − 9) and *m/z* 179.1609/203.1609 (n − 7) (Fig. 6b). The spectra disclosed two double bond positional isomers, WE(20:0–18:1(11)) and WE (20:0–18:1(9)). Wax esters identified in vernix caseosa using MS^2^ UVPD and MS^3^ CID/UVPD are listed in Table S2 and Table S3, respectively.

## Conclusions

Activation of lithium adducts of wax esters with laser radiation at 213 nm provides structurally informative mass spectra. Photofragments in the MS^2^ UVPD spectra make it possible to determine the lengths of the acid and alcohol chains and the numbers and positions of double bonds in the chains. Norrish and Norrish-Yang photochemical reactions are likely involved after the absorption of UV photons by the ester moiety. Among the fragments, lithiated aldehyde ions related to acid and alcohol chains are found; they are abundant in spectra of saturated wax esters and wax esters with few double bonds. The alcohol chain-related and acid chain-related aldehyde fragments can be distinguished by their satellite ions. Fragments consistent with lithiated fatty acids are found in the spectra as well. Their intensity increases with the increasing number of double bonds in wax esters; they are the most intense fragments of polyunsaturated wax esters. Aldehyde and acid fragments are well suited for determining aliphatic chain lengths and the number of double bonds in these chains. Absorption of UV photons to double bonds provides fragments useful for deducing double bond positions within the aliphatic chains. The double bond in monounsaturated wax esters manifests itself by an easily recognizable pair of fragments differing by 24.000 Da. The number of double bond-related fragments increases if there are more double bonds in the wax ester molecule. In the case of methylene-interrupted double bonds, all C=C and C−C bonds are cleaved, creating characteristic patterns of fragments. The position of these patterns on the *m/z* axis determines the position of the methylene-interrupted double bond system.

For the interpretation of UVPD spectra, an analyzer with high resolving power and mass accuracy is advantageous. We used Orbitrap, which operated at a resolution of 120,000. The intensity of photofragments increased with increasing activation time. The relative ion intensities initially varied but stabilized and remained almost constant at higher activation times. Good quality spectra were obtained using activation times longer than 100 ms; a value of 500 ms was used to generate spectra of standards. Comparative experiments with sodium, potassium, and ammonium ions showed that the UVPD of wax esters works best when the esters are in the form of lithium adducts.

The ability of ESI MS^2^ UVPD to identify wax esters in natural samples was tested for two commonly used approaches in lipidomic analysis. Jojoba oil wax esters were separated by reversed-phase UHPLC using a mobile phase flow rate of 180 µl/min. The chromatographic peak widths were about 0.4 min. Using an activation time of 700 ms, about 20 MS^2^ UVPD spectra were collected across the peaks. To demonstrate the applicability of UHPLC ESI MS^2^ UVPD for analyzing wax ester mixtures, the most abundant wax esters were unambiguously identified in the jojoba oil sample. Direct infusion or shotgun analysis was the second approach tested for wax esters. Selected lithiated wax ester peaks from the vernix caseosa sample were fragmented by MS^2^ UVPD or MS^3 ^CID/UVPD. The MS^2^ demonstrates a better sensitivity than MS^3^; however, the interpretation of MS^2^ UVPD spectra may not be entirely conclusive, particularly in the case of complex samples. For instance, if the precursor ion represents a mixture of unsaturated wax esters differing by the position of the ester group, double bond positions cannot be linked to individual fatty acyls. In such situations, MS^3^ CID/UVPD helps to refine structural information. Dedicated software tools would make UVPD data evaluation more effective.

We can summarize that the ESI MS^2^ UVPD is an excellent tool for the structural analysis of wax esters for samples directly introduced into the ion source or separated by UHPLC. Its strength is, in particular, the ability to identify the positions of double bonds in the aliphatic chains of wax esters. Robust data interpretation workflows can be achieved by combining UVPD and CID (HCD) steps in parallel or serial arrangement.

## Supplementary Information

Below is the link to the electronic supplementary material.

Supplementary file1 (DOCX 9698 KB)
