# Supplementary material for: Structural characterization of wax esters using ultraviolet photodissociation mass spectrometry
Source: Anal Bioanal Chem. 2024 Jul 20;416(25):5497–512. doi: 10.1007/s00216-024-05434-2 (PMC11427557; doi:10.1007/s00216-024-05434-2)
Supplement: Supplementary file 1 — Supplementary file1 (DOCX 9698 KB) [file 216_2024_5434_MOESM1_ESM.docx]

**Supplementary Information**

**Structural Characterization of Wax Esters Using Ultraviolet Photodissociation Mass Spectrometry**

Barbora Kloudová, Vladimír Vrkoslav, Miroslav Polášek, Zuzana Bosáková, Josef Cvačka

**List of Contents:**

| Table S1: Wax ester standards . . . . . . . . . . . . . . . . . . . . . . . . . . . . . . . . . . . . . . . . . . . . . . . . . . . . . . | 2 |
| --- | --- |
| Text S1: UVPD spectra of different adducts of wax esters . . . . . . . . . . . . . . . . . . . . . . . . . . . . . . . . | 3 |
| Figure S1: MS^2^ UVPD spectra of [M + Na]^+^ and [M + K]^+^ of WE(16:0/14:1(9Z)) . . . . . . . . . . . . . . | 4 |
| Figure S2: EI spectrum of WE(16:0/14:1(9Z)) . . . . . . . . . . . . . . . . . . . . . . . . . . . . . . . . . . . . . . . . . . | 5 |
| Figure S3: MS^2^ UVPD spectra of [M + NH_4_]^+^ and [M + H]^+^ of WE 16:0/14:1(9Z) . . . . . . . . . . . . . | 6 |
| Text S2: UVPD activation time . . . . . . . . . . . . . . . . . . . . . . . . . . . . . . . . . . . . . . . . . . . . . . . . . . . . . | 7 |
| Figure S4: Survival yield curves for WE(16:0/14:1(9Z)) and WE(12:0/18:2(9Z,12Z)) . . . . . . . . . . | 8 |
| Figure S5: Activation time-resolved curves (relative abundance) for WE(16:0/14:1(9Z)) . . . . . . . . | 9 |
| Figure S6: Activation time-resolved curves (relative abundance) for WE(12:0/18:2(9Z,12Z)) . . . . . | 10 |
| Figure S7: Activation time-resolved curves (ion fraction) for WE(16:0/14:1(9Z)) . . . . . . . . . . . . . . | 11 |
| Figure S8: Activation time-resolved curves (ion fraction) for WE(12:0/18:2(9Z,12Z)) . . . . . . . . . . | 12 |
| Figure S9: Sections of MS^2^ UVPD spectra of [M + Li]^+^ WE(12:0/18:2(9Z,12Z)) . . . . . . . . . . . . . . | 13 |
| Scheme S-I: Proposed UVPD fragmentation pathways: Norrish type I . . . . . . . . . . . . . . . . . . . . . . . | 14 |
| Scheme S-II: Proposed UVPD fragmentation pathways: Norrish type II . . . . . . . . . . . . . . . . . . . . . . | 14 |
| Figure S10: MS^2^ CID and MS^2^ HCD spectra of [M + Li]^+^ of WE(12:0/22:0) . . . . . . . . . . . . . . . . . . | 15 |
| Figure S11: MS^2^ UVPD spectrum of [M + Li]^+^ of WE(12:0/18:0) . . . . . . . . . . . . . . . . . . . . . . . . . . | 16 |
| Figure S12: MS^2^ UVPD spectrum of [M + Li]^+^ of WE(14:0/14:0) . . . . . . . . . . . . . . . . . . . . . . . . . . | 17 |
| Scheme S-III: Formation of a pair of fragments identifying double bond position . . . . . . . . . . . . . . | 17 |
| Figure S13: MS^2^ CID and MS^2^ HCD spectra of [M + Li]^+^ of WE(16:0/14:1(9Z)) . . . . . . . . . . . . . . . | 18 |
| Figure S14: MS^2^ CID and MS^3^ CID/UVPD spectra of [M + Li]^+^ of WE(18:1(9Z)/14:1(9Z)) . . . . . . | 19 |
| Figure S15: MS^2^ UVPD spectra of [M + Li]^+^ of WE(20:0/18:2(9Z,12Z)), WE(22:0/18:2(9Z,12Z)) | 20 |
| Figure S16: MS^2^ UVPD spectrum of [M + Li]^+^ of WE(12:0/20:4(5Z, 8Z,11Z,14Z)) . . . . . . . . . . . . | 21 |
| Figure S17: Sections of MS^2^ UVPD spectra of *m/z* 595 from jojoba oil . . . . . . . . . . . . . . . . . . . . . . | 22 |
| Figure S18: Full scan spectrum of lithium adducts of lipids from vernix caseosa . . . . . . . . . . . . . . . | 23 |
| Table S2: Wax esters identified in vernix caseosa by ESI MS^2^ UVPD . . . . . . . . . . . . . . . . . . . . . . . | 24 |
| Table S3: Wax esters identified in vernix caseosa by ESI MS^3^ CID/UVPD . . . . . . . . . . . . . . . . . . . | 30 |
| SI References: . . . . . . . . . . . . . . . . . . . . . . . . . . . . . . . . . . . . . . . . . . . . . . . . . . . . . . . . . . . . . . . . . . . | 30 |

**Table S1**. Wax esters standards.

| **Wax ester** | **Shorthand notation** |
| --- | --- |
| Lauryl stearate | WE(12:0/18:0) |
| Lauryl linoleate | WE(12:0/18:2(9Z,12Z)) |
| Lauryl arachidonate | WE(12:0/20:4(5Z, 8Z,11Z,14Z)) |
| Lauryl behenate | WE(12:0/22:0) |
| Myristyl myristate | WE(14:0/14:0) |
| Palmityl myristoleate | WE(16:0/14:1(9Z)) |
| Stearyl laurate | WE(18:0/12:0) |
| Stearyl oleate | WE(18:0/18:1(9Z)) |
| Oleyl myristoleate | WE(18:1(9Z)/14:1(9Z)) |
| Oleyl stearate | WE(18:1(9Z)/18:0) |
| Arachidyl linoleate | WE(20:0/18:2(9Z,12Z)) |
| Arachidyl arachidonate | WE(20:0/20:4(5Z, 8Z,11Z,14Z)) |
| Behenyl linoleate | WE(22:0/18:2(9Z,12Z)) |
| Behenyl α-linolenate | WE(22:0/18:3(9Z,12Z,15Z)) |

## **Text S1**. UVPD spectra of different adducts of wax esters

Previous research has shown that the UVPD of lithium adducts makes it possible to determine the positions of double bonds in aliphatic chains of lipids. We were interested in whether other types of adducts could also be used for these purposes. Therefore, we compared MS^2^ UVPD spectra of Li^+^, Na^+^, K^+^, H^+^, and NH_4_^+^ adducts of WE(16:0/14:1(9Z)). The lithium adduct provided two fragments separated by the mass of two carbon atoms at *m/z* 399.3793 (C_26_H_48_LiO_2_^+^) and *m/z* 375.3793 (C_24_H_48_LiO_2_^+^), clearly indicating the n-5 double bond (Figure 2a). Other fragments made it possible to characterize alcohol and acid aliphatic chains, as described in detail in the Results and Discussion Section. The photoactivation of sodium adduct provided acid- and alcohol-related aldehyde fragments *m/z* 233.1870 (C_14_H_26_ONa^+^) and *m/z* 263.2340 (C_16_H_32_ONa^+^), respectively. Except for these ions, the fragmentation of the sodium adduct (Figure S1a) differed significantly from the lithium adduct. Neutral loss of sodium was an important fragmentation channel, leading to radical cation C13H25COOC16H33+• at *m/z* 450.4421. The radical cation fragmented further, giving ions that can also be observed in the EI spectrum (Figure S2). The most abundant ions were C13H25C(OH)OH+ at *m/z* 227.1999 and C16H33OCO+ at *m/z* 269.2469. The fragmentation of the potassium adduct (Figure S1b) resembled that of the sodium adduct, with the aldehyde fragments being significantly less abundant. In addition to the neutral loss of potassium, there was a significant hydrogen loss from [M + K]^+^. The fragments describing cleavages at the double bond site were low in the spectrum of sodium adduct and completely absent in the potassium adduct spectrum. For completeness, MS^2^ UVPD spectra of [M + H]^+^ and [M + NH_4_]^+^ were recorded as well (Figure S3). Neither of these precursors yielded fragments useful for determining double bond positions. The results showed that lithium adducts are best suited for determining the positions of double bonds in unsaturated wax esters using UVPD.


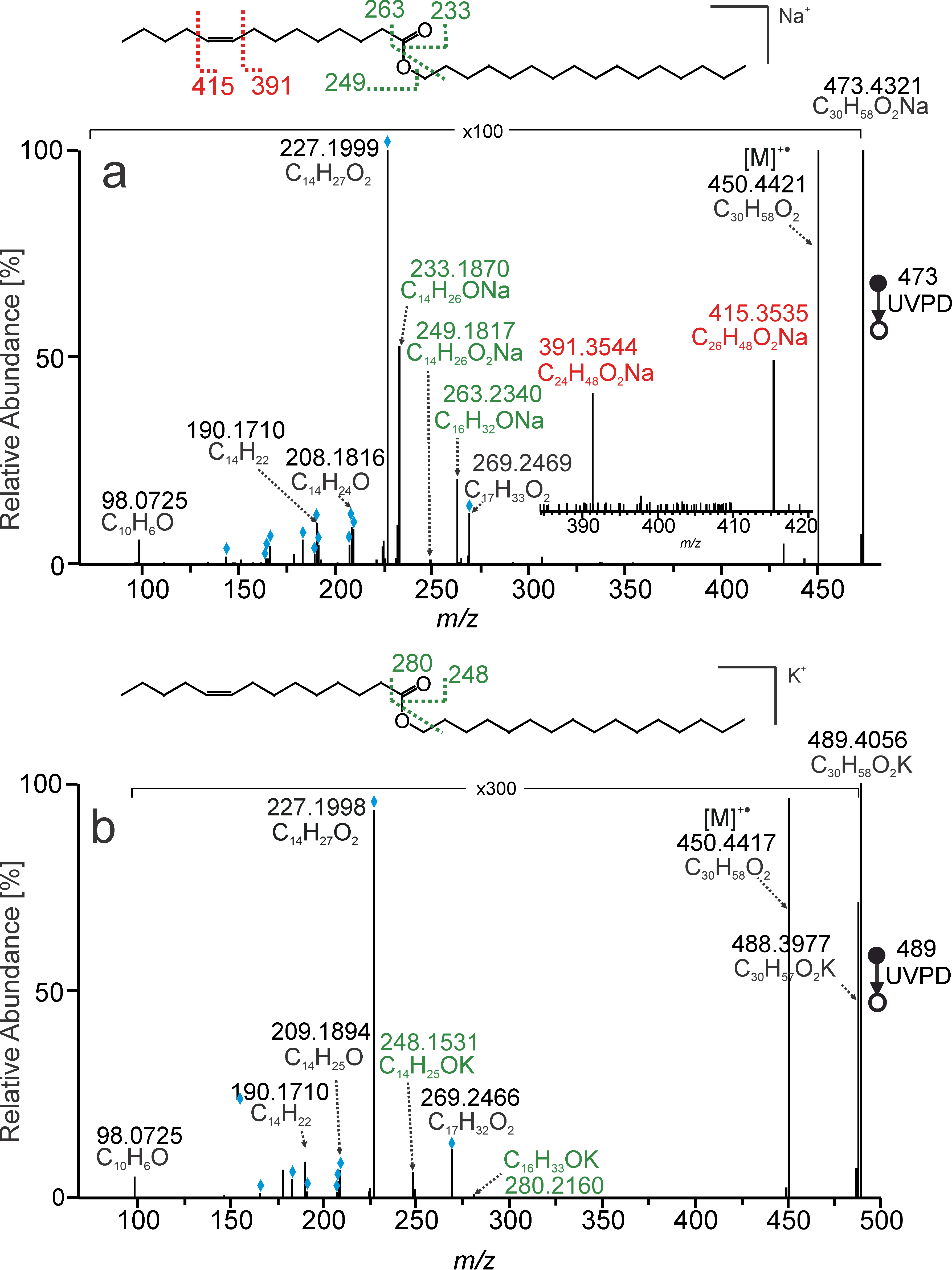


**Figure S1.** MS^2^ UVPD spectra of [M + Na]^+^ (a) and [M + K]^+^ (b) of palmityl myristoleate ((WE(16:0/14:1(9Z)))) recorded using the activation time of 500 ms. Cationized aldehyde and fatty acid fragments are highlighted in green, and the pair of fragments indicating the position of the double bond is marked in red. Ions formed by C13H25COOC16H33+• fragmentations are marked with a blue symbol.


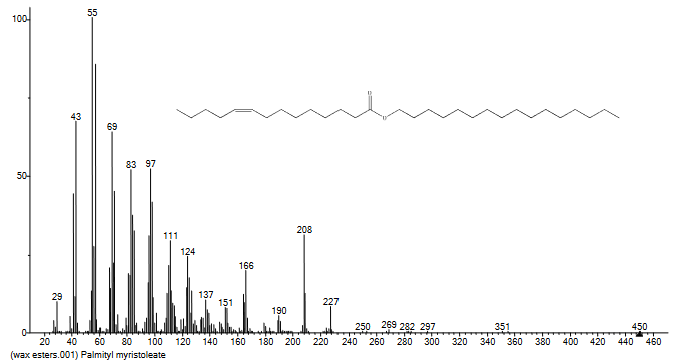


**Figure S2**. EI spectrum of palmityl myristoleate WE(16:0/14:1(9Z)) (ref. S1).


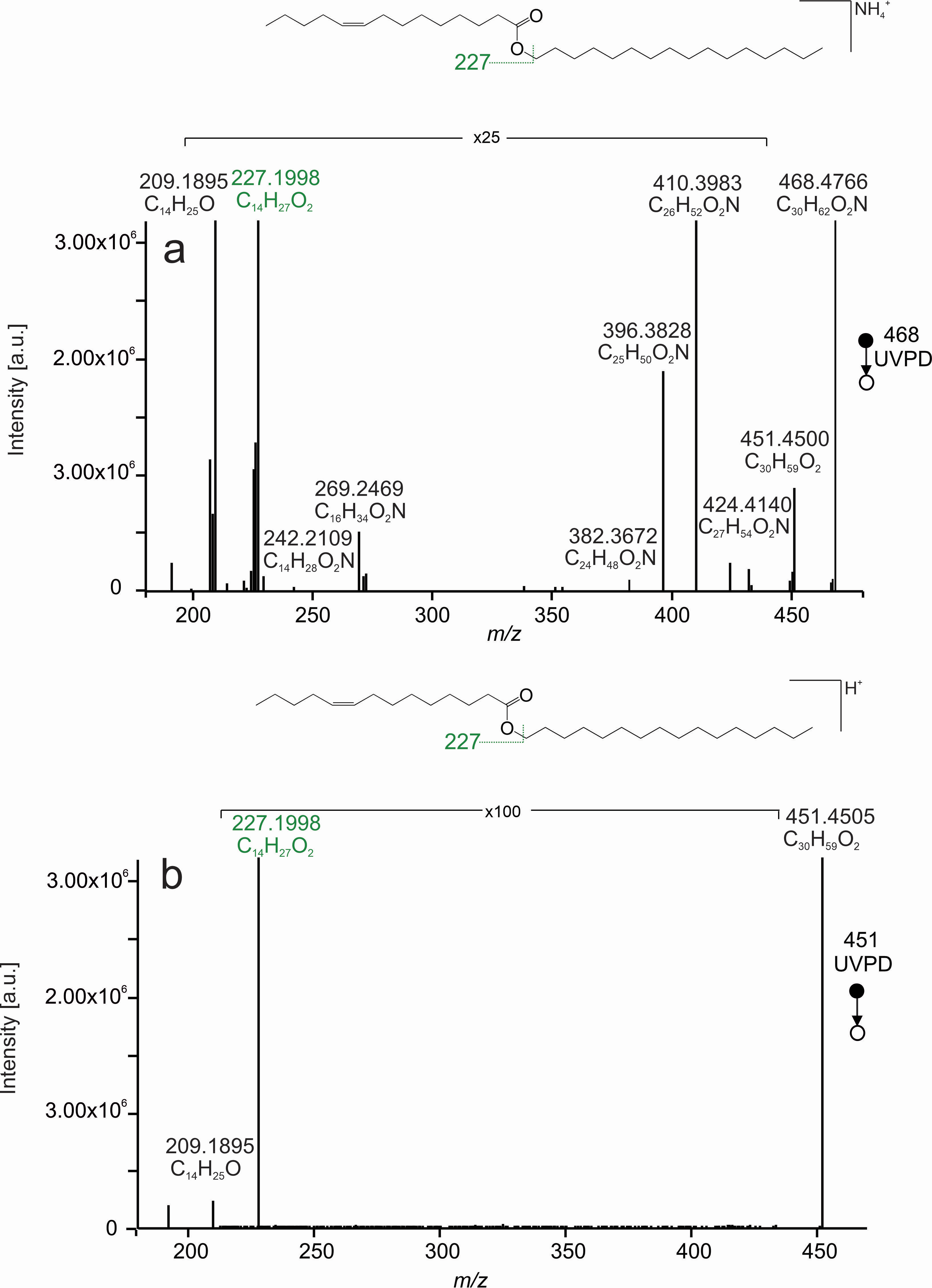


**Figure S3**. MS^2^ UVPD spectrum of [M + NH_4_]^+^ (a) and [M + H]^+^ (b) of palmityl myristoleate WE(16:0/14:1(9Z)). A fragment corresponding to protonated myristoleic acid is indicated in green.

**Text S2**. *UVPD activation time*

To understand the effect of activation time on wax esters, mass spectra of two standards, WE(16:0/14:1(9Z)) and WE(12:0/18:2(9Z,12Z)), were recorded using 10 to 4000 milliseconds activation times. The photodissociation efficiency was expressed using the residual [M + Li]^+^ intensity ratio to the sum of all ion intensities (survival yield). For both standards, survival yield decreased linearly with increasing activation time (Figure S4). The decomposition of the precursor ion having two double bonds was significantly faster compared to the wax ester with one double bond; after 4000 ms, the survival yield of [M + Li]^+^ was 96% for WE(16:0/14:1(9Z)) and 67% for WE(12:0/18:2(9Z,12Z)). The abundance of the fragments increased with increasing activation time (Figure S5, S6). Figures S7 and S8 show the effect of activation time on the proportions of fragment ions. The relative ion intensities initially varied but stabilized and remained almost constant at higher activation times. Diagnostic ions describing the wax ester aliphatic chains were detectable already after 25 ms. However, fragments diagnostic for double bonds required a longer activation, at least 100 ms. Importantly, the whole pattern of diagnostic ions for double bonds did not change with further increasing the activation time (Figure S9). The results suggest the possibility of using long activation times for increasing fragment intensities (e.g., in infusion experiments) without negative consequences for the spectra quality.

**Figure S4**. Activation time-resolved curves showing survival yield values for [M + Li]^+^ of WE(16:0/14:1(9Z)), *m/z* 457.5 and WE(12:0/18:2(9Z,12Z)), *m/z* 455.5. The survival yield was calculated as the ratio of the intensity of the precursor to the sum of the intensities of the precursor and all fragments in the spectrum.

**Figure S5**. Activation time-resolved curves showing relative abundances of fragment ions in MS^2^ UVPD spectra of lithium adduct of WE(16:0/14:1(9Z)). The relative abundances are the values read on the y-axis in the spectra (calculated by dividing the ion count of the fragment by the ion count of the spectrum base peak, i.e., unfragmented precursor ion, and multiplied by 100).

**Figure S6.** Activation time-resolved curves showing relative abundances of fragment ions in MS^2^ UVPD spectra of lithium adduct of WE(12:0/18:2(9Z,12Z)). The relative abundances are the values read on the y-axis in the spectra (calculated by dividing the ion count of the fragment by the ion count of the spectrum base peak, i.e., unfragmented precursor ion, and multiplied by 100).

**Figure S7**. Activation time-resolved curves showing fractions of fragment ions in MS^2^ UVPD spectra of [M + Li]^+^ of WE(16:0/14:1(9Z)). The main diagnostic fragments: acid-related aldehyde *m/z* 217.2, alcohol-related aldehyde *m/z* 247.2, pair of the double bond-related fragments *m/z* 375.4, *m/z* 399.4. The ion fraction was calculated as the ratio of the intensity of the fragment ion to the sum of the intensities of all fragments in the spectrum.

**

**Figure S8**. Activation time-resolved curves showing fractions of fragment ions in MS^2^ UVPD spectra of [M + Li]^+^ of WE(12:0/18:2(9Z,12Z)). The main diagnostic fragments: lithiated fatty acid *m/z* 287.3, acid-related aldehyde *m/z* 271.3, double bond-related fragments *m/z* 345.3, *m/z* 357.3. The ion fraction was calculated as the ratio of the intensity of the fragment ion to the sum of the intensities of all fragments in the spectrum.


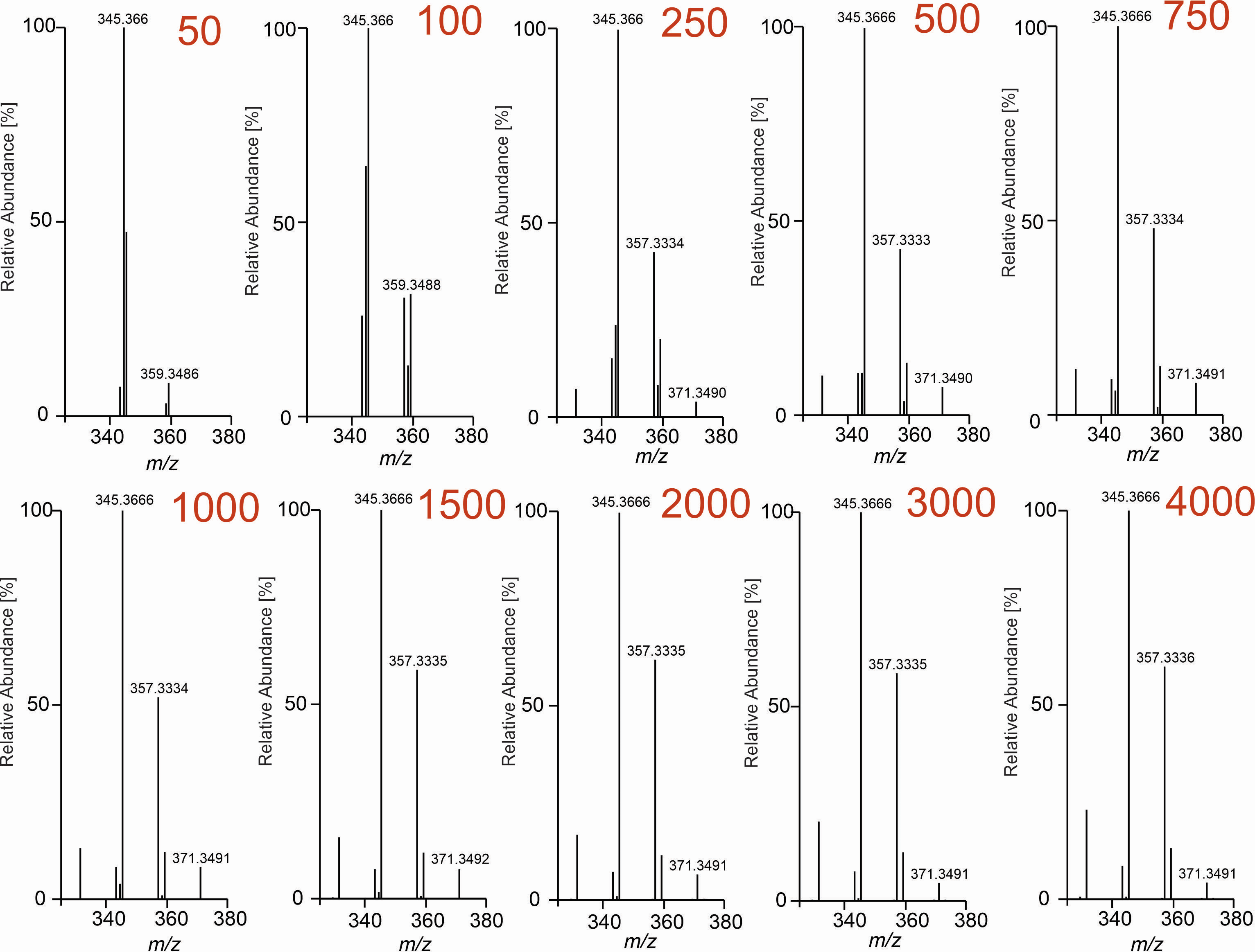


**Figure S9.** Sections of MS^2^ UVPD spectra of the lithium adduct of WE(12:0/18:2(9Z,12Z)) showing fragments related to double bond cleavages. Indicated activation times are in milliseconds.

**Scheme S-I**. Proposed UVPD fragmentation pathways: Norrish type I photochemical cleavage of WE(12:0/22:0) (a) and a secondary reaction with a hydrogen transfer yielding lithiated ketene and lithiated alcohol (b).

**Scheme S-II**. Proposed UVPD fragmentation pathways: Norrish type II photochemical cleavage of WE(12:0/22:0) (a) and cleavage of disubstituted oxetan-2-ol formed by Norrish-Yang photochemical cyclization of WE(12:0/22:0) (b). In both mechanisms, lithiated behenic acid is formed.


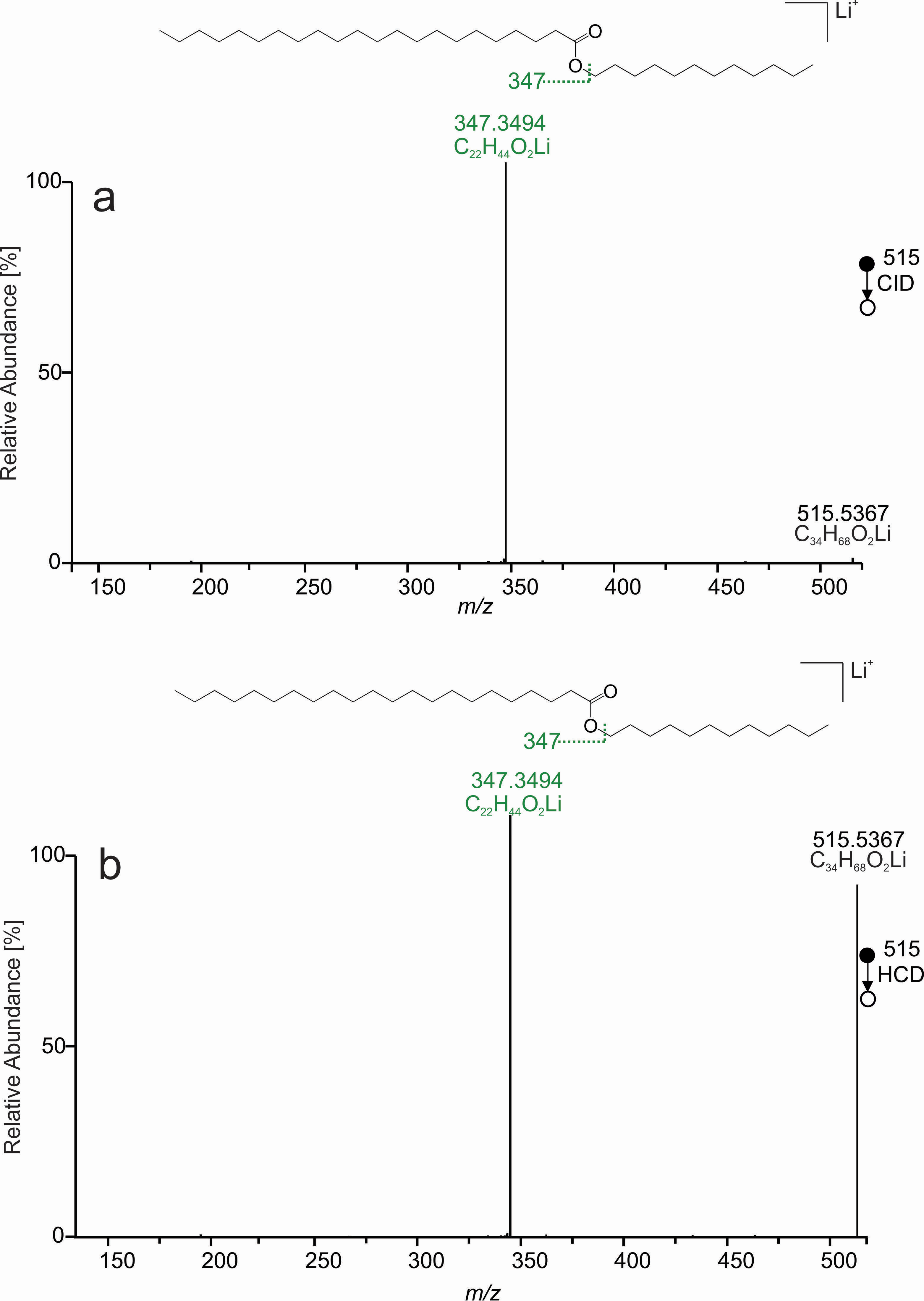


**Figure S10**. MS^2^ CID (a) and MS^2^ HCD (b) spectra of [M + Li]^+^ of lauryl behenate WE(12:0/22:0) recorded using NCE 30% (CID) and 40% (HCD).


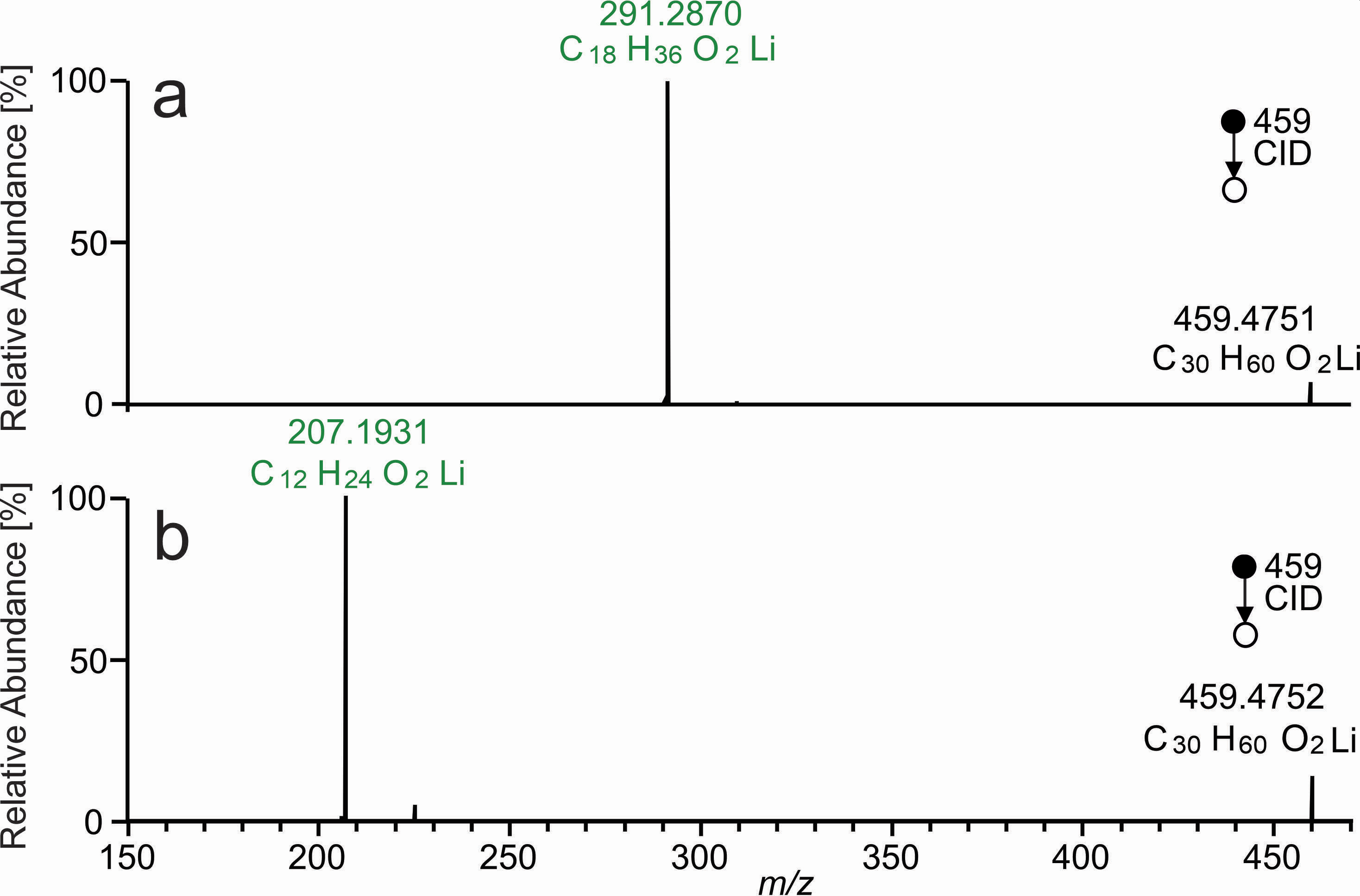


**Figure S11**. MS^2^ CID spectra of [M + Li]^+^ of lauryl stearate WE(12:0/18:0) (a) and stearyl laurate WE(18:0/12:0) (b) recorded using NCE 40%. The main fragments correspond to lithiated stearic acid *m/z* 291.3 (a) and lithiated lauric acid *m/z* 207.2 (b).


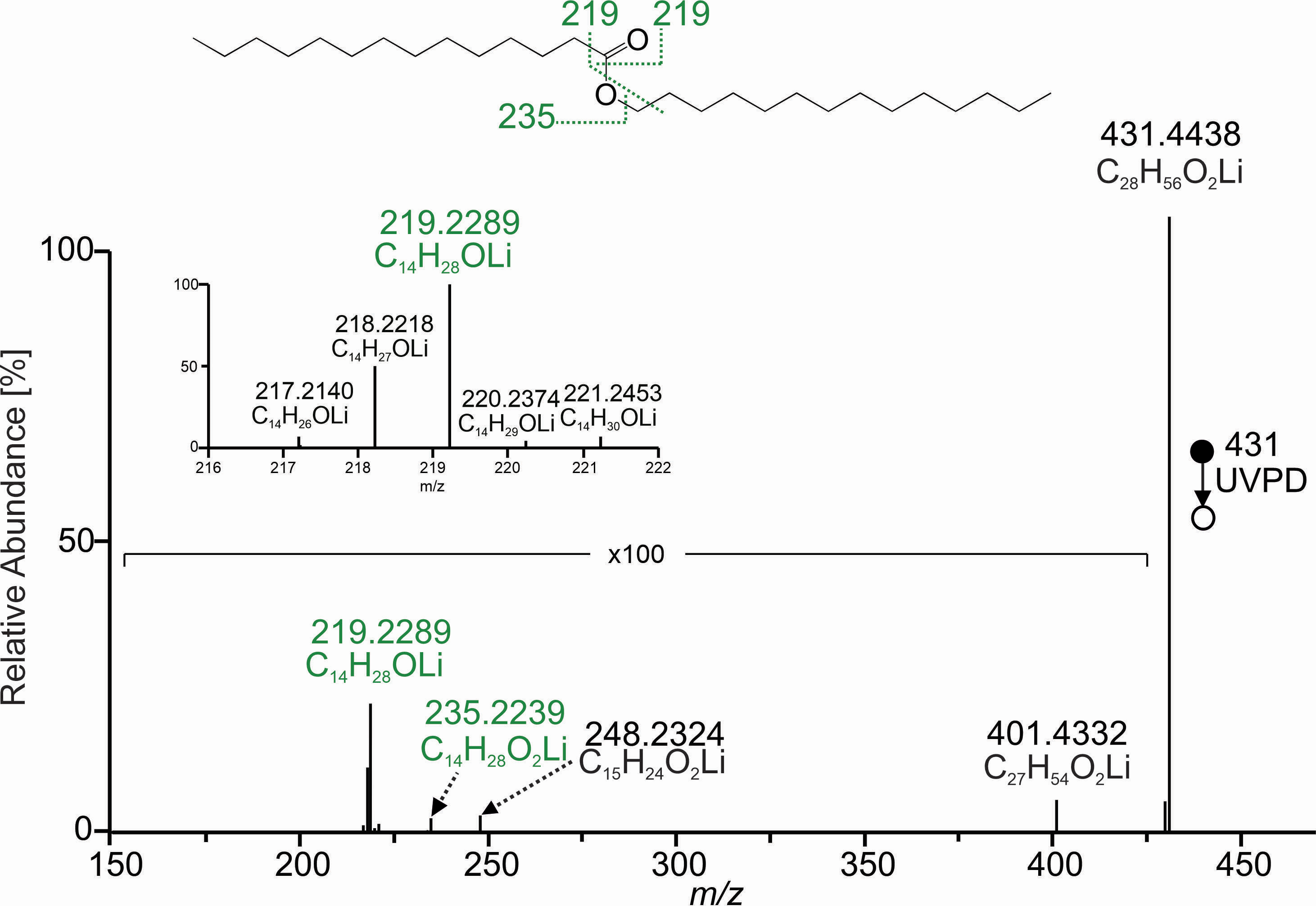


**Figure S12**. MS^2^ UVPD spectrum of [M + Li]^+^ of myristyl myristate WE(14:0/14:0) recorded using the activation time of 500 ms.

**Scheme S-III**. Formation of a pair of diagnostic fragments indicating the position of the double bond (ref. S2).

*
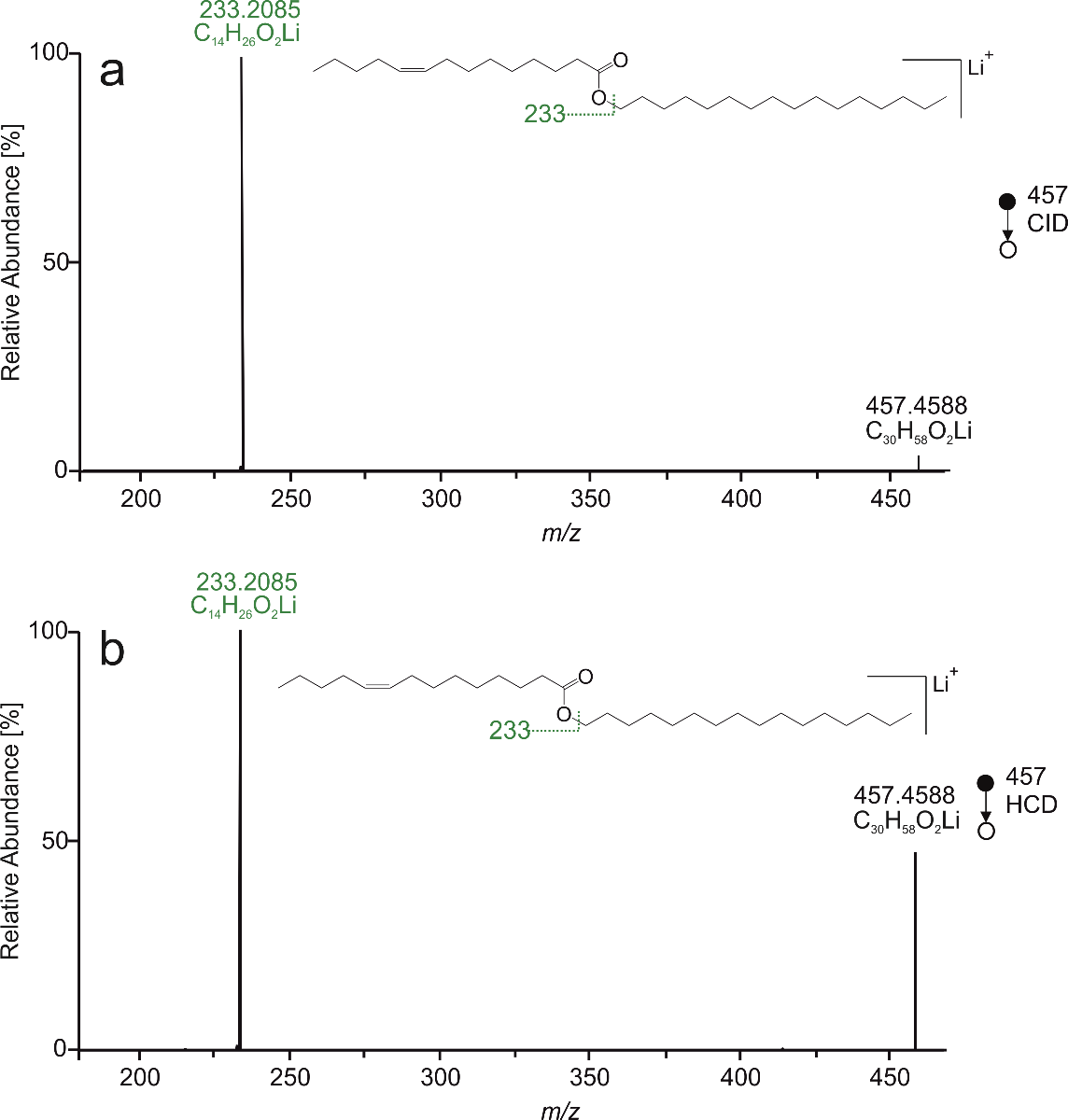
*

**Figure S13**. MS^2^ CID (a) MS^2^ HCD (b) spectra of [M+Li]^+^ of palmityl myristoleate WE(16:0/14:1(9Z)) recorded using NCE 30% (CID) and 40% (HCD).


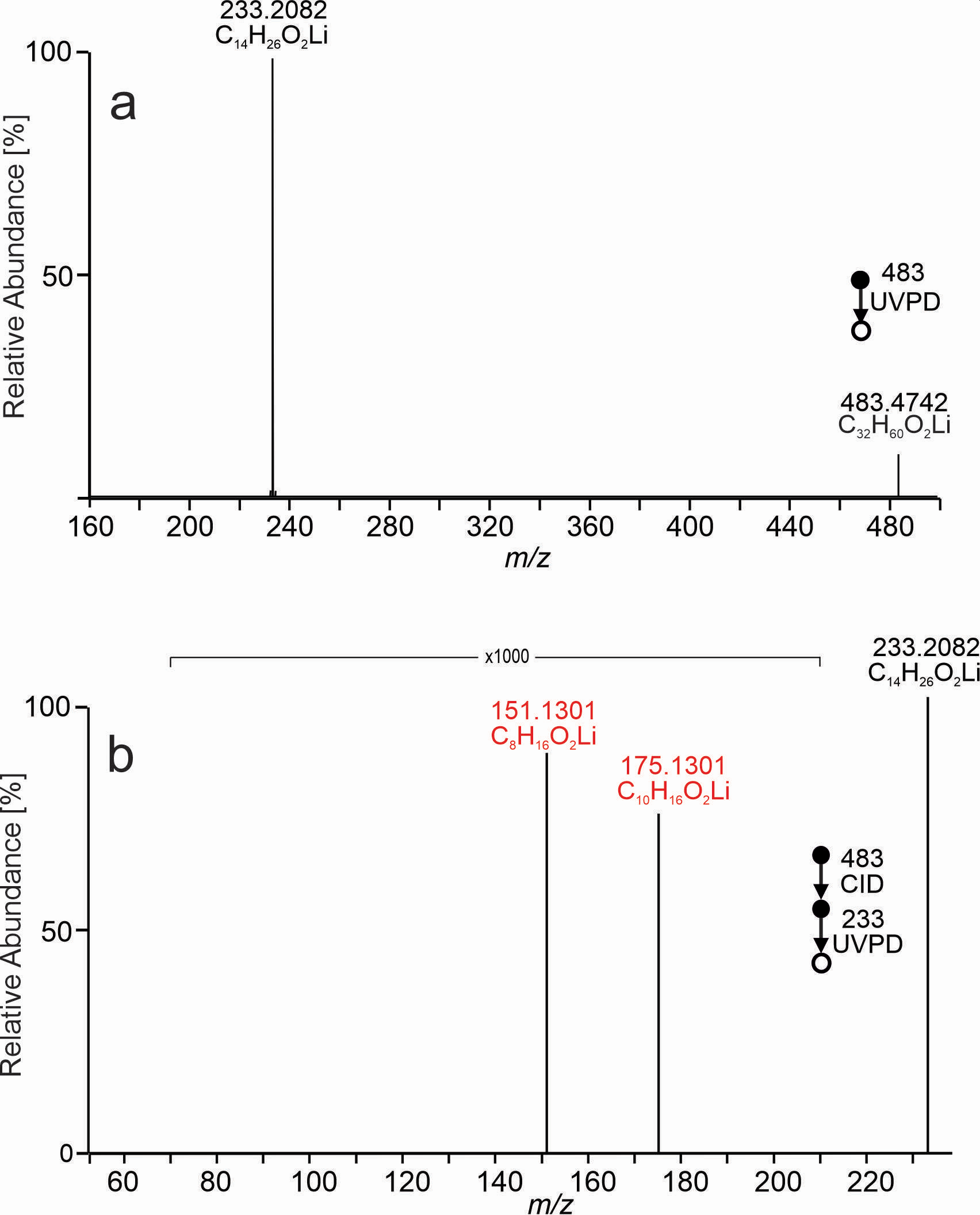


**Figure S14**. MS^2^ CID (a) and MS^3^ CID/UVPD (b) spectra of [M + Li]^+^ of oleyl myristoleate WE(18:1(9Z)/14:1(9Z)) recorded using NCE 40% (CID) and UVPD activation time of 500 ms.

**
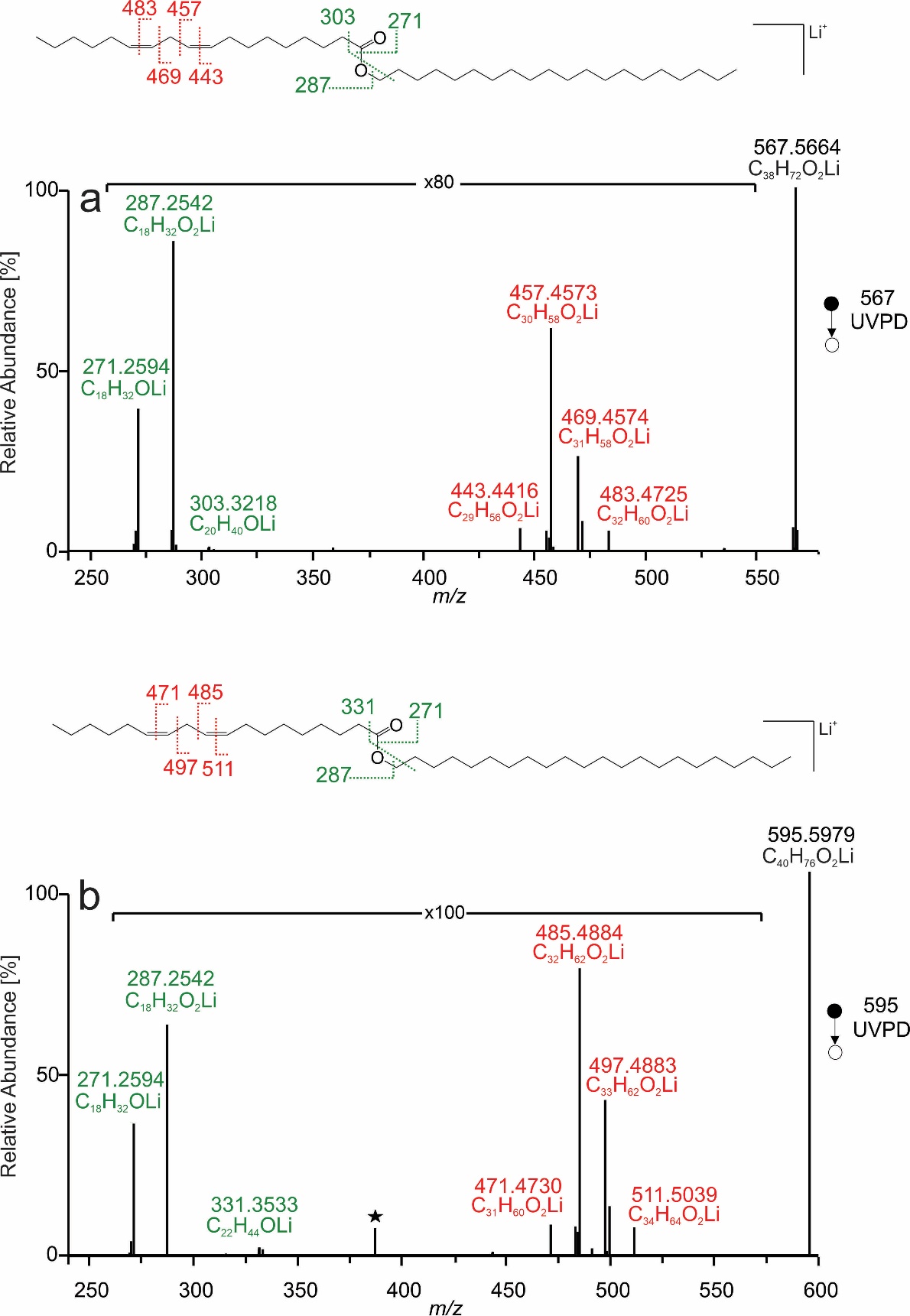
**

**Figure S15**. MS^2^ UVPD spectra of [M + Li]^+^ of arachidyl linoleate WE(20:0/18:2(9Z,12Z)) (a) and behenyl linoleate WE(22:0/18:2(9Z,12Z)) (b) recorded using the activation time of 500 ms.


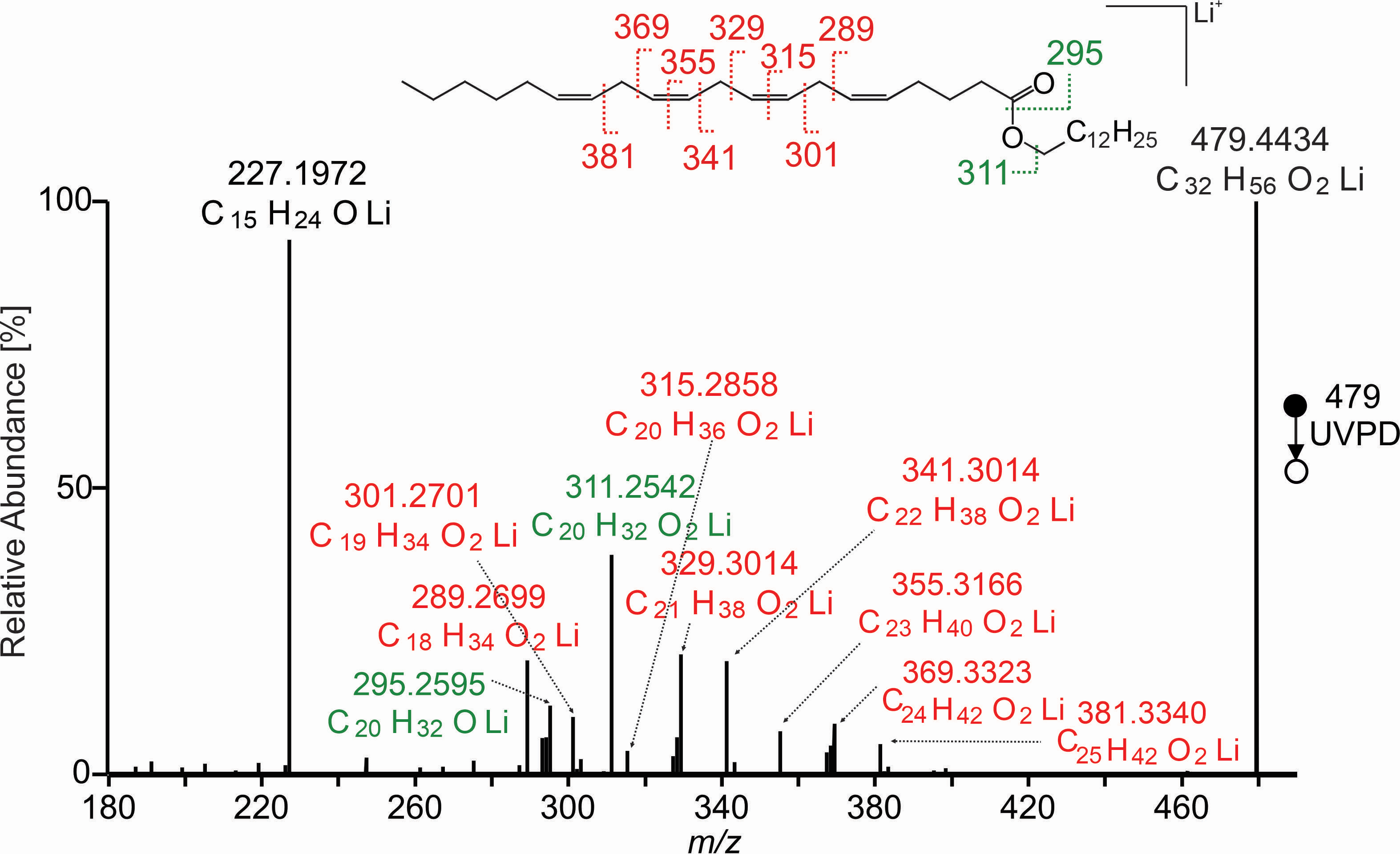


**Figure S16**. MS^2^ UVPD spectra of [M + Li]^+^ of lauryl arachidonate WE(12:0/20:4(5Z, 8Z,11Z,14Z)) recorded using the activation time of 500 ms.


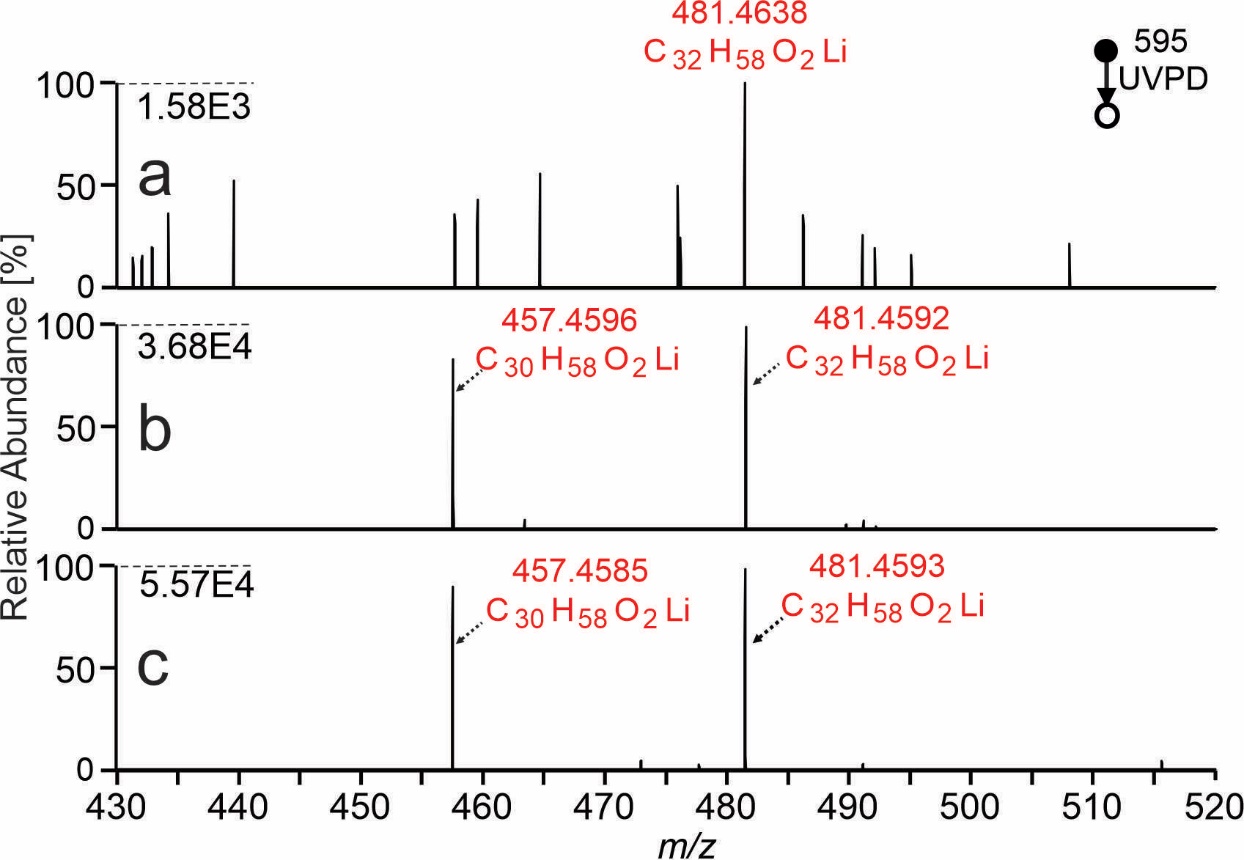


**Figure S17**. Sections of MS^2^ UVPD spectra of *m/z* 595 from jojoba oil sample showing the effect of activation time on the spectra quality. The spectra show the double bond-related pair of fragments *m/z* 457/481 recorded with activation times of 100 ms (a), 500 ms (b), and 700 ms (c).


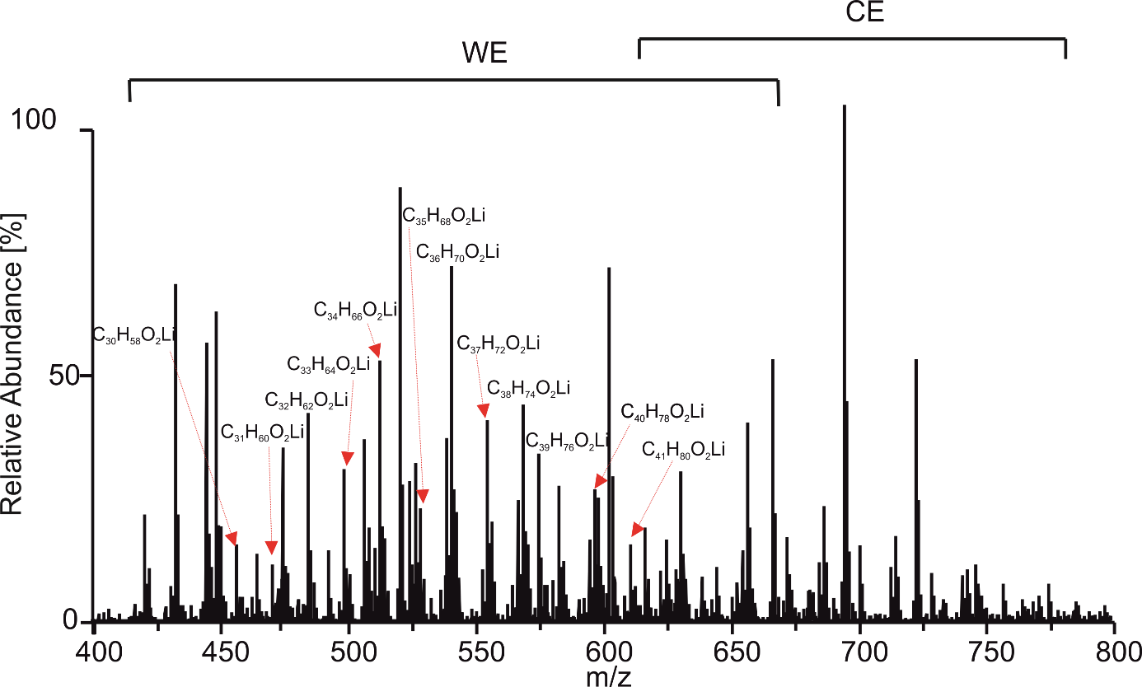


**Figure S18**. Full scan mass spectrum of lithium adducts of lipids isolated from vernix caseosa. WE – wax esters; CE – cholesteryl esters.

**Table S2**. Wax esters identified in vernix caseosa by ESI MS^2^ UVPD.

| Precursor ion | Acid ions | Aldehyde ions | Diagnostic pair of ions for double bond localization | Identification | Double bond position |
| --- | --- | --- | --- | --- | --- |
| 457.4581 | 261.2391-C_16_H_30_O_2_Li  233.2080-C_14_H_26_O_2_Li | 217.2131-C_14_H_26_OLi  219.2286-C_14_H_28_OLi 245.2444-C_16_H_30_OLi  247.2600-C_16_H_32_OLi | 305.3018/329.3018(C_19_H_38_O_2_Li/C_21_H_38_O_2_Li)  333.3331/357.3331(C_21_H_42_O_2_Li/C_23_H_42_O_2_Li)  347.3487/371.3485(C_22_H_44_O_2_Li/C_24_H_44_O_2_Li) | 14:0/16:1  16:0/14:1 | n−7  n−8  n−10 |
| 471.4731 | 233.2081-C_14_H_26_O_2_Li  247.2235-C_15_H_28_O_2_Li  261.2391-C_16_H_30_O_2_Li  263.2548-C_16_H_32_O_2_Li  275.2547-C_17_H_32_O_2_Li | 217.2131-C_14_H_26_OLi  219.2286-C_14_H_28_OLi  231.2286-C_15_H_28_OLi  233.244-C_15_H_30_OLi  245.2444-C_16_H_30_OLi  247.2599-C_16_H_32_OLi  259.2598-C_17_H_32_OLi 261.2755-C_17_H_34_OLi | 319.3173/343.3174(C_20_H_40_O_2_Li/C_22_H_40_O_2_Li)  333.3331/357.3331(C_21_H_42_O_2_Li/C_23_H_42_O_2_Li)  347.3484/371.3485(C_22_H_44_O_2_Li/C_24_H_44_O_2_Li) | 17:0/14:1  15:0/16:1  16:0/15:1  14:0/17:1  15:1/16:0 | n−10  n−9  n−8 |
| 499.5045 | 233.2080-C_14_H_26_O_2_Li  247.2234-C_15_H_28_O_2_Li  261.2391-C_16_H_30_O_2_Li  275.2546-C_17_H_32_O_2_Li  289.2702-C_18_H_34_O_2_Li | 217.2131-C_14_H_26_OLi  231.2285-C_15_H_28_OLi  233.2443-C_15_H_30_OLi  245.2441-C_16_H_30_OLi  247.2234-C_16_H_32_OLi  259.2597-C_17_H_32_OLi  261.2754-C_17_H_34_OLi  273.2754-C_18_H_34_OLi  275.2911-C_18_H_36_OLi  289.3067-C_19_H_38_OLi | 333.3331/357.3331(C_21_H_42_O_2_Li/C_23_H_42_O_2_Li)  347.3484/371.3485(C_22_H_44_O_2_Li/C_24_H_44_O_2_Li)  361.3640/385.3639(C_23_H_46_O_2_Li/C_25_H_46_O_2_Li)  375.3795/399.3797(C_24_H_48_O_2_Li/C_26_H_48_O_2_Li) | 19:0/14:1  17:0/16:1  18:0/15:1  16:0/17:1  15:0/18:1 | n−8  n−9  n−10  n−11 |
| 513.5201 | 233.2081-C_14_H_26_O_2_Li  247.2234-C_15_H_28_O_2_Li  261.2391-C_16_H_30_O_2_Li  289.2702-C_18_H_34_O_2_Li  317.3013-C_20_H_38_O_2_Li | 217.2130-C_14_H_26_OLi  219.2286-C_14_H_28_OLi  231.2285-C_15_H_28_OLi  245.2441-C_16_H_30_OLi  247.2599-C_16_H_32_OLi  273.2754-C_18_H_34_OLi  275.2711-C_18_H_36_OLi  289.3067-C_19_H_38_OLi  301.3066-C_20_H_38_OLi  303.3222-C_20_H_40_OLi | 347.3483/371.3485(C_22_H_44_O_2_Li/C_24_H_44_O_2_Li)  361.3640/385.3639(C_23_H_46_O_2_Li/C_25_H_46_O_2_Li)  375.3795/399.3797(C_24_H_48_O_2_Li/C_26_H_48_O_2_Li)  389.3953/413.3952(C_25_H_50_O_2_Li/C_27_H_50_O_2_Li  403.4106/427.4112(C_26_H_52_O_2_Li/C_28_H_52_O_2_Li) | 20:0/14:1  14:0/20:1  19:0/15:1  18:0/16:1  16:0/18:1 | n−11  n−10  n−9  n−8  n−7 |
| 527.5359 | 233.2081-C_14_H_26_O_2_Li  247.2234-C_15_H_28_O_2_Li  261.2391-C_16_H_30_O_2_Li  275.2546-C_17_H_32_O_2_Li  289.2702-C_18_H_34_O_2_Li | 217.2130-C_14_H_26_OLi  231.2285-C_15_H_28_OLi  245.2441-C_16_H_30_OLi  259.2598-C_17_H_32_OLi  261.2754-C_17_H_34_OLi  273.2754-C_18_H_34_OLi  275.2711-C_18_H_36_OLi  289.3067-C_19_H_38_OLi  303.3227-C_20_H_40_OLi  317.3379-C_21_H_42_OLi | 347.3483/371.3485(C_22_H_44_O_2_Li/C_24_H_44_O_2_Li)  361.3640/385.3639(C_23_H_46_O_2_Li/C_25_H_46_O_2_Li)  375.3795/399.3797(C_24_H_48_O_2_Li/C_26_H_48_O_2_Li)  403.4106/427.4112(C_26_H_52_O_2_Li/C_28_H_52_O_2_Li)  389.3953/413.3952(C_25_H_50_O_2_Li/C_27_H_50_O_2_Li)  417.4263/441.4265(C_27_H_54_O_2_Li/C_29_H_54_O_2_Li) | 21:0/14:1  20:0/15:1  19:0/16:1  17:0/18:1  18:0/17:1 | n−12  n−11  n−10  n−9  n−8  n−7 |
| 541.5518 | 247.2234-C_15_H_28_O_2_Li  261.2391-C_16_H_30_O_2_Li  263.2349-C_16_H_32_O_2_Li  289.2702-C_18_H_34_O_2_Li | 231.2285-C_15_H_28_OLi  245.2445-C_16_H_30_OLi  247.2551-C_16_H_32_OLi  273.2754-C_18_H_34_OLi  275.2919-C_18_H_36_OLi 301.3070-C_20_H_38_OLi  303.3227-C_20_H_40_OLi | 389.3953/413.3952(C_25_H_50_O_2_Li/C_27_H_50_O_2_Li)  403.4106/427.4112(C_26_H_52_O_2_Li/C_28_H_52_O_2_Li) | 21:0/15:1  20:0/16:1  20:1/16:0  18:0/18:1 | n−10  n−9 |
| 555.5670 | 247.2234-C_15_H_28_O_2_Li  249.2393-C_15_H_30_O_2_Li  261.2393-C_16_H_30_O_2_Li  263.2548-C_16_H_32_O_2_Li  275.2546-C_17_H_32_O_2_Li  289.2704-C_18_H_34_O_2_Li  319.3218-C_20_H_38_O_2_Li | 231.2285-C_15_H_28_OLi  233.2443-C_15_H_30_OLi  245.2445-C_16_H_30_OLi  247.2551-C_16_H_32_OLi  259.2598-C_17_H_32_OLi  261.2754-C_17_H_34_OLi  273.2754-C_18_H_34_OLi  289.3068-C_19_H_38_OLi 301.3070-C_20_H_40_OLi  303.3226-C_20_H_38_OLi  315.2694-C_21_H_40_OLi  317.3383-C_21_H_42_OLi  331.3534-C_22_H_44_OLi | 389.3953/413.3952(C_25_H_50_O_2_Li/C_27_H_50_O_2_Li)  375.3795/399.3797(C_24_H_48_O_2_Li/C_26_H_48_O_2_Li)  403.4106/427.4112(C_26_H_52_O_2_Li/C_28_H_52_O_2_Li)  417.4263/441.4265(C_27_H_54_O_2_Li/C_29_H_54_O_2_Li)  431.4421/455.4426(C_28_H_56_O_2_Li/C_30_H_56_O_2_Li) | 22:0/15:1  21:0/16:1  21:1/16:0  20:0/17:1  17:0/20:1  19:0/18:1 | n−12  n−11  n−10  n−9  n−8 |
| 569.5827 | 233.2081-C_14_H_26_O_2_Li  261.2390-C_16_H_30_O_2_Li  263.2548-C_16_H_32_O_2_Li  289.2704-C_18_H_34_O_2_Li  317.3014-C_20_H_38_O_2_Li  319.3218-C_20_H_40_O_2_Li  345.3328-C_22_H_42_O_2_Li  347.3481-C_22_H_44_O_2_Li | 217.2130-C_14_H_26_OLi  245.2445-C_16_H_30_OLi  247.2551-C_16_H_32_OLi  273.2754-C_18_H_34_OLi  275.2919-C_18_H_36_OLi  301.3065-C_20_H_38_OLi  303.3070-C_20_H_40_OLi 329.3377-C_22_H_42_OLi  331.3534-C_22_H_44_OLi  359.3851-C_24_H_48_OLi | 389.3953/413.3952(C_25_H_50_O_2_Li/C_27_H_50_O_2_Li)  403.4106/427.4112(C_26_H_52_O_2_Li/C_28_H_52_O_2_Li)  417.4263/441.4265(C_27_H_54_O_2_Li/C_29_H_54_O_2_Li)  431.4421/455.4426(C_28_H_56_O_2_Li/C_30_H_56_O_2_Li)  459.4731/483.4731(C_30_H_60_O_2_Li/C_32_H_60_O_2_L)  483.4731/511.5033(C_32_H_64_O_2_Li/C_34_H_64_O_2_Li) | 24:0/14:1  22:1/16:0  22:0/16:1  16:0/22:1  16:1/22:0  20:0/18:1  18:1/20:0  18:0/20:1 | n−12  n−11  n−10  n−9  n−7  n−5 |
| 583.5981 | 247.2234-C_15_H_28_O_2_Li  249.2393-C_15_H_30_O_2_Li  261.2393-C_16_H_30_O_2_Li  263.2548-C_16_H_32_O_2_Li  275.2547-C_17_H_32_O_2_Li  289.2704-C_18_H_34_O_2_Li  317.3014-C_20_H_38_O_2_Li  373.3641-C_24_H_46_O_2_Li  375.3796-C_24_H_48_O_2_Li | 231.2285-C_15_H_28_OLi  233.2443-C_15_H_30_OLi  245.2445-C_16_H_30_OLi  247.2551-C_16_H_32_OLi  259.2598-C_17_H_32_OLi  273.2754-C_18_H_34_OLi  287.2910-C_19_H_38_OLi  303.3226-C_20_H_38_OLi  317.3379-C_21_H_42_OLi  331.3536-C_22_H_44_OLi  345.3692-C_23_H_46_OLi  357.3692-C_24_H_46_OLi 359.3851-C_24_H_48_OLi | 389.3953/413.3952(C_25_H_50_O_2_Li/C_27_H_50_O_2_Li)  403.4106/427.4112(C_26_H_52_O_2_Li/C_28_H_52_O_2_Li)  417.4263/441.4265(C_27_H_54_O_2_Li/C_29_H_54_O_2_Li)  431.4421/455.4426(C_28_H_56_O_2_Li/C_30_H_56_O_2_Li)  459.4731/483.4731(C_30_H_60_O_2_Li/C_32_H_60_O_2_L)  473.4888/497.4887(C_31_H_62_O_2_Li/C_33_H_62_O_2_Li) | 15:0/24:1  15:1/24:0  24:1/15:0  24:0/15:1  23:0/16:1  22:0/17:1  21:0/18:1  19:0/20:1 | n−13  n−12  n−11  n−10  n−8  n−7 |
| 597.6135 | 233.2081-C_14_H_26_O_2_Li  235.2235-C_14_H_28_O_2_Li  247.2234-C_15_H_28_O_2_Li  261.2393-C_16_H_30_O_2_Li  275.2547-C_17_H_32_O_2_Li  289.2704-C_18_H_34_O_2_Li  317.3014-C_20_H_38_O_2_Li  373.3641-C_24_H_46_O_2_Li  375.3796-C_24_H_48_O_2_Li | 217.2131-C_14_H_26_OLi  219.2286-C_14_H_28_OLi  231.2285-C_15_H_28_OLi  245.2445-C_16_H_30_OLi  247.2551-C_16_H_32_OLi  259.2598-C_17_H_32_OLi  273.2754-C_18_H_34_OLi  301.3070-C_20_H_38_OLi  303.3226-C_20_H_40_OLi  331.3536-C_22_H_44_OLi  345.3692-C_23_H_46_OLi  359.3851-C_24_H_48_OLi  373.4003-C_25_H_50_OLi  385.4003-C_26_H_50_OLi  387.4159-C_26_H_52_OLi | 403.4106/427.4112(C_26_H_52_O_2_Li/C_28_H_52_O_2_Li)  417.4263/441.4265(C_27_H_54_O_2_Li/C_29_H_54_O_2_Li)  431.4421/455.4426(C_28_H_56_O_2_Li/C_30_H_56_O_2_Li)  445.4574/469.4574(C_29_H_58_O_2_Li/C_31_H_58_O_2_Li)  459.47.30/483.47.28(C_30_H_60_O_2_Li/C_32_H_60_O_2_Li)  473.4888/497.4887(C_31_H_62_O_2_Li/C_33_H_62_O_2_Li)  483.4731/511.5033(C_32_H_64_O_2_Li/C_34_H_64_O_2_Li) | 26:1/14:0  26:0/14:1  25:0/15:1  24:0/16:1  23:0/17:1  22:0/18:1  16:0/24:1  16:1/24:0  20:0/20:1 | n−13  n−12  n−11  n−10  n−9  n−8  n−7 |
| 611.6290 | 247.2234-C_15_H_28_O_2_Li  249.2393-C_15_H_30_O_2_Li  261.2393-C_16_H_30_O_2_Li  275.2547-C_17_H_32_O_2_Li  277.2703-C_17_H_34_O_2_Li  289.2704-C_18_H_34_O_2_Li | 231.2285-C_15_H_28_OLi  233.2443-C_15_H_30_OLi  245.2445-C_16_H_30_OLi  259.2598-C_17_H_32_OLi  261.2754-C_17_H_34_OLi  273.2754-C_18_H_34_OLi  345.3692-C_23_H_46_OLi  357.3692-C_24_H_46_OLi  359.3851-C_24_H_48_OLi  373.4003-C_25_H_50_OLi  385.4003-C_26_H_50_OLi  387.4159-C_26_H_52_OLi | 417.4263/441.4265(C_27_H_54_O_2_Li/C_29_H_54_O_2_Li)  445.4574/469.4574(C_29_H_58_O_2_Li/C_31_H_58_O_2_Li)  459.47.30/483.4728(C_30_H_60_O_2_Li/C_32_H_60_O_2_L)  473.4887/497.4887(C_31_H_62_O_2_Li/C_33_H_62_O_2_Li)  487.5043/511.5041(C_32_H_64_O_2_Li/C_34_H_64_O_2_Li)  501.5199/525.5198(C_33_H_66_O_2_Li/C_35_H_66_O_2_Li) | 26:0/15:1  26:1/15:0  25:0/16:1  24:0/17:1  24:1/17:0  23:0/18:1 | n−13  n−11  n−10  n−9  n−8  n−7 |
| 625.6443 | 261.2393-C_16_H_30_O_2_Li  263.2346-C_16_H_32_O_2_Li  275.2547-C_17_H_32_O_2_Li  289.2704-C_18_H_34_O_2_Li  317.3014-C_20_H_38_O_2_Li  345.3328-C_22_H_42_O_2_Li  347.3487-C_22_H_44_O_2_Li | 245.2445-C_16_H_30_OLi  247.2551-C_16_H_32_OLi  259.2598-C_17_H_32_OLi  273.2754-C_18_H_34_OLi  301.3070-C_20_H_38_OLi  303.3226-C_20_H_40_OLi  329.3377-C_22_H_42_OLi  331.3536-C_22_H_44_OLi  359.3851-C_24_H_48_OLi  373.4003-C_25_H_50_OLi  385.4003-C_26_H_50_OLi  387.4159-C_26_H_52_OLi | 473.4887/497.4887(C_31_H_62_O_2_Li/C_33_H_62_O_2_Li)  459.47.30/483.47.28(C_30_H_60_O_2_Li/C_32_H_60_O_2_L)  487.5043/511.5041(C_32_H_64_O_2_Li/C_34_H_64_O_2_Li)  501.5199/525.5198(C_33_H_66_O_2_Li/C_35_H_66_O_2_Li)  515.5353/539.5354(C_34_H_68_O_2_Li/C_36_H_68_O_2_Li) | 26:0/16:1  26:1/16:0  25:0/17:1  24:0/18:1  22:0/20:1  20:1/22:0  20:0/22:1 | n−11  n−10  n−9  n−8  n−7 |
| 639.6607 | 249.2393-C_15_H_30_O_2_Li  261.2393-C_16_H_30_O_2_Li  275.2547-C_17_H_32_O_2_Li  289.2704-C_18_H_34_O_2_Li  401.3954-C_26_H_50_O_2_Li  431.4420-C_28_H_56_O_2_Li | 231.2285-C_15_H_28_OLi  233.2443-C_15_H_30_OLi  245.2445-C_16_H_30_OLi  259.2598-C_17_H_32_OLi  261.2754-C_17_H_34_OLi  273.2754-C_18_H_34_OLi  373.4003-C_25_H_50_OLi  385.4003-C_26_H_50_OLi  387.4159-C_26_H_52_OLi  401.4318-C_27_H_54_OLi  413.4317-C_28_H_54_OLi  415.4478-C_28_H_56_OLi | 445.4574/469.4574(C_29_H_58_O_2_Li/C_31_H_58_O_2_Li)  473.4887/497.4887(C_31_H_62_O_2_Li/C_33_H_62_O_2_Li)  487.5043/511.5041(C_32_H_64_O_2_Li/C_34_H_64_O_2_Li)  501.5199/525.5198(C_33_H_66_O_2_Li/C_35_H_66_O_2_Li) | 28:1/15:0  15:1/28:0  27:0/16:1  26:0/17:1  25:0/18:1  17:0/26:1 | n−13  n−11  n−10  n−9 |
| 653.6761 | 233.2081-C_14_H_26_O_2_Li  235.235-C_14_H_28_O_2_Li  261.2393-C_16_H_30_O_2_Li  275.2547-C_17_H_32_O_2_Li  289.2704-C_18_H_34_O_2_Li  401.3954-C_26_H_50_O_2_Li  431.4420-C_28_H_56_O_2_Li | 217.2131-C_14_H_26_OLi  219.2286-C_14_H_28_OLi  245.2445-C_16_H_30_OLi  259.2598-C_17_H_32_OLi  273.2754-C_18_H_34_OLi  275.2919-C_18_H_36_OLi  385.4003-C_26_H_50_OLi  387.4159-C_26_H_52_OLi  401.4318-C_27_H_54_OLi  415.4478-C_28_H_56_OLi  441.4629-C_30_H_58_OLi  443.4786-C_30_H_60_OLi | 501.5199/525.5198(C_33_H_66_O_2_Li/C_35_H_66_O_2_Li)  487.5043/511.5041(C_32_H_64_O_2_Li/C_34_H_64_O_2_Li)  515.5353/539.5354(C_34_H_68_O_2_Li/C_36_H_68_O_2_Li)  525.5201/553.5513(C_35_H_70_O_2_Li/C_37_H_70_O_2_Li)  543.5671/567.3671(C_36_H_72_O_2_Li/C_38_H_72_O_2_Li) | 30:0/14:1  30:1/14:0  28:0/16:1  27:0/17:1  26:0/18:1  16:1/28:0  18:0/26:1 | n−11  n−10  n−9  n−8  n−7 |

**Table S3**. Wax esters identified in vernix caseosa by ESI MS^3^ CID/UVPD.

| *Precursor ion for MS^2^* | *Precursor ion for MS^3^* | *Diagnostic pair of ions for double bond localization* | *Identification* |
| --- | --- | --- | --- |
| 555.5800 | 261.2391 | 109.0833/133.0832 (C_5_H_10_O_2_Li/C_7_H_10_O_2_Li) | 21:0/16:1(n−10) |
|  | 275.2546 | 109.0833/133.0832 (C_5_H_10_O_2_Li/C_7_H_10_O_2_Li)  123.0988/147.0986 (C_6_H_12_O_2_Li/C_8_H_12_O_2_Li) | 20:0/17:1(n−11)  20:0/17:1(n−10) |
| 569.5825 | 261.2391 | 109.0833/133.0832 (C_5_H_10_O_2_Li/C_7_H_10_O_2_Li)  123.0988/147.0986 (C_6_H_12_O_2_Li/C_8_H_12_O_2_Li)  151.1299/175.1299 (C_8_H_16_O_2_Li/C_10_H_16_O_2_Li) | 22:0/16:1(n−10)  22:0/16:1(n−9)  22:0/16:1(n−7) |
|  | 289.2703 | 151.1299/175.1299 (C_8_H_16_O_2_Li/C_10_H_16_O_2_Li)  179.1611/203.1609 (C_10_H_20_O_2_Li/C_12_H_20_O_2_Li) | 20:0/18:1(n−9)  20:0/18:1(n−7) |

**SI References**

**S1**. Urbanová K, Vrkoslav V, Valterová I, Háková M, Cvačka J. Structural characterization of wax esters by electron ionization mass spectrometry. J Lipid Res. 2012; 53:204-213.

**S2**. Ryan E, Nguyen CQN, Shiea C, Reid GE. Detailed Structural Characterization of Sphingolipids via 193 nm Ultraviolet Photodissociation and Ultra High Resolution Tandem Mass Spectrometry. J Am Soc Mass Spectrom. 2017; 28:1406–1419.
